# Analyses of human vaccine-specific circulating and bone marrow-resident B cell populations reveal benefit of delayed vaccine booster dosing with blood-stage malaria antigens

**DOI:** 10.3389/fimmu.2023.1193079

**Published:** 2024-01-17

**Authors:** Jordan R. Barrett, Sarah E. Silk, Catherine G. Mkindi, Karolina M. Kwiatkowska, Mimi M. Hou, Amelia M. Lias, Wilmina F. Kalinga, Ivanny M. Mtaka, Kirsty McHugh, Martino Bardelli, Hannah Davies, Lloyd D. W. King, Nick J. Edwards, Virander S. Chauhan, Paushali Mukherjee, Stella Rwezaula, Chetan E. Chitnis, Ally I. Olotu, Angela M. Minassian, Simon J. Draper, Carolyn M. Nielsen

**Affiliations:** ^1^Department of Biochemistry, University of Oxford, Oxford, United Kingdom; ^2^Jenner Institute, University of Oxford, Oxford, United Kingdom; ^3^Ifakara Health Institute, Bagamoyo, Tanzania; ^4^International Centre for Genetic Engineering and Biotechnology (ICGEB), New Delhi, India; ^5^Multi Vaccines Development Program (MVDP), New Delhi, India; ^6^Muhimbili National Hospital, Dar es Salaam, Tanzania; ^7^Unité de Biologie de Plasmodium et Vaccins, Institut Pasteur, Université Paris Cité, Paris, France

**Keywords:** B cells, vaccine dosing regimen, malaria, memory B cells, plasma cells, bone marrow, antibody, T cells

## Abstract

We have previously reported primary endpoints of a clinical trial testing two vaccine platforms for the delivery of *Plasmodium vivax* malaria DBPRII: viral vectors (ChAd63, MVA), and protein/adjuvant (PvDBPII with 50µg Matrix-M™ adjuvant). Delayed boosting was necessitated due to trial halts during the pandemic and provides an opportunity to investigate the impact of dosing regimens. Here, using flow cytometry – including agnostic definition of B cell populations with the clustering tool CITRUS – we report enhanced induction of DBPRII-specific plasma cell and memory B cell responses in protein/adjuvant versus viral vector vaccinees. Within protein/adjuvant groups, delayed boosting further improved B cell immunogenicity compared to a monthly boosting regimen. Consistent with this, delayed boosting also drove more durable anti-DBPRII serum IgG. In an independent vaccine clinical trial with the *P. falciparum* malaria RH5.1 protein/adjuvant (50µg Matrix-M™) vaccine candidate, we similarly observed enhanced circulating B cell responses in vaccinees receiving a delayed final booster. Notably, a higher frequency of vaccine-specific (putatively long-lived) plasma cells was detected in the bone marrow of these delayed boosting vaccinees by ELISPOT and correlated strongly with serum IgG. Finally, following controlled human malaria infection with *P. vivax* parasites in the DBPRII trial, *in vivo* growth inhibition was observed to correlate with DBPRII-specific B cell and serum IgG responses. In contrast, the CD4+ and CD8+ T cell responses were impacted by vaccine platform but not dosing regimen and did not correlate with *in vivo* growth inhibition in a challenge model. Taken together, our DBPRII and RH5 data suggest an opportunity for protein/adjuvant dosing regimen optimisation in the context of rational vaccine development against pathogens where protection is antibody-mediated.

## Introduction

The roll-out of various SARS-CoV-2 vaccines during the COVID-19 pandemic highlighted the importance of understanding the immunological significance of booster dose timing in order to maximise protective efficacy. Increased peak serum antibody concentrations were observed with delayed booster regimens in both viral vector (1, 2) and mRNA (3) delivery platforms, indicating potential opportunities to maximise humoral immunity through the optimisation of antigen delivery timing. The impact of delayed booster dosing regimens on peak responses has also been explored in more depth by earlier work from the *Plasmodium falciparum* malaria field with both the blood-stage malaria vaccine candidate RH5 (4, 5) and the pre-erythrocytic vaccine candidate, RTS,S [now WHO-approved as Mosquirix (6–10)]. Notably, the impact of delayed booster dosing on vaccine-specific serum IgG *durability* has been published less widely; the RH5 trials remain the only example of improvements in durability through modifications in booster dosing regimens (4, 5). We have previously speculated on the underlying biological mechanisms and reasons for discrepancies between the RTS,S and RH5 trials (5), and these questions require further investigation. Furthermore, it is critical to confirm whether the delayed booster phenomenon is a broader immunological principle, or an anomaly restricted to the RH5.1/AS01 vaccine candidate.

Here, we present analyses of B cell, T cell, and serum antibody responses to a blood-stage malaria antigen from a different species of malaria – DBPRII, *Plasmodium vivax* – and investigate the impact of delayed boosters (**Table 1**). This work builds on the initial clinical trial publication, which focused on the efficacy results of the controlled human malaria infection (CHMI) (11). Supporting these findings, we additionally present peripheral B cell analyses (using the same B cell flow cytometry panel) from a *P. falciparum* RH5.1/Matrix-M™ vaccine trial, where PBMC samples were available for exploratory analysis. For the first time in the context of malaria vaccinology, we also report on vaccine-specific bone marrow plasma cells.

**Table 1:** Vaccination regimens in DBPRII and RH5 clinical trials.

Target	Platform	Dosing regimen	Timing of vaccinations (m = month)				*n* ^$^
			Dose 1	Dose 2	Dose 3	Dose 4	
*P. vivax* DBPRII	ChAd63-DBPRII and MVA-DBPRII viral vectors	Monthly [VV-M]	0m	2m	–		6
		Delayed [VV-D]	0m	17m	19m		2
	PvDBPII protein/Matrix-M™	Monthly [PA-M]	0m	1m	2m		4
		Delayed [PA-D]	0m	1m	14m		8
		Delayed + booster [PA-DB]	*0m*	*1m*	*14m*	19m	5
			*(Same vaccinees as PA-D)*				
*P. falciparum* RH5	RH5.1 protein/Matrix-M™	Monthly [M]	0m	1m	2m		5
		Delayed [D]	0m	1m	6m*		6

Post-vaccination samples analysed in this study are following the final vaccination (FV), indicated by the highlighted cell. See Methods for further details of clinical trial design.

^$^ Reflects sample size of groups completing full vaccination regimen in clinical trials (11). All trial participants were healthy adults. Sample sizes for individual assays are specified in figure legends. To note, sample sizes preclude head-to-head comparisons of VV-M versus VV-D.

* Final dose of antigen (but not adjuvant) in the delayed arm of *P. falciparum* RH5 trial was also fractionated (see Methods).

Taken together, our data support delayed booster protein/adjuvant vaccination in the next phase of *P. vivax* DBPRII and *P. falciparum* RH5 vaccine development and, more broadly, support the consideration of alternative dosing regimens for vaccines against a range of pathogens where protection is antibody-mediated.

## Results

### Delayed protein/adjuvant booster dosing increases the induction of circulating DBPRII- and RH5-specific plasma cells and memory B cells

**Figure 1:**
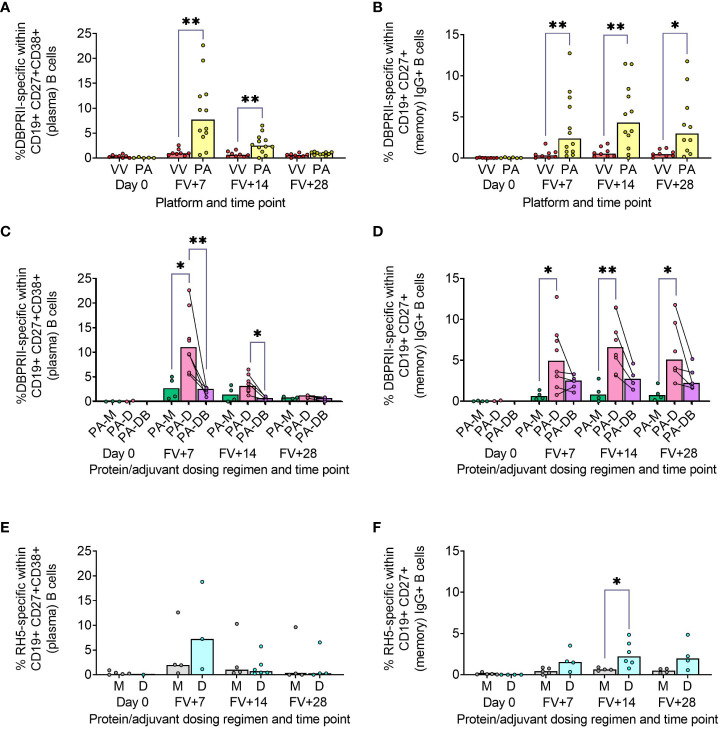
Vaccine-specific plasma cell and memory IgG+ B cell responses. PBMC from pre-vaccination (Day 0) and post-final vaccination (FV) time points were analysed for B cell responses by flow cytometry; gating strategies are as described in Methods and **Supplementary Figures 1** and **3**. Frequencies of DBPRII-specific B cells – identified by probe staining – were compared between vaccine platforms (A, B) or protein/adjuvant dosing regimens (C, D) within both plasma cell (A, C) or memory IgG+ B cell (B, D) populations. Similarly, frequencies of RH5-specific B cells were compared between protein/adjuvant dosing regimens within plasma cells (E) and memory IgG+ B cells (F). IgM+, IgA+, activated and resting memory B cell responses are shown in **Supplementary Figures 2** and **4**. VV = ChAd63-MVA viral vectors [monthly and delayed dosing]; PA = PvDBPII protein/adjuvant [PA-M and PA-D]; PA-M = PvDBPII protein/adjuvant monthly dosing; PA-D = PvDBPII protein/adjuvant delayed booster dosing; PA-DB = PvDBPII protein/adjuvant delayed booster dosing with extra booster; M = RH5.1/adjuvant monthly dosing; D = RH5.1/adjuvant delayed booster dosing. Post-vaccination comparisons were performed between DBPRII platforms (A, B) or RH5 dosing regimens (E, F) with Mann-Whitney U tests or between PvDBPII protein/adjuvant dosing regimens by Kruskal Wallis test with Dunn’s correction for multiple comparisons (C, D). Sample sizes for all assays were based on sample availability; each circle represents a single sample. (A, B) VV/PA: Day 0 = 8/5-6, FV+7 = 8/12, FV+14 = 8/12, FV+28 = 8/10. (C, D) PA-M/PA-D/PA-DB: Day 0 = 3-4/2/na, FV+7 = 4/8/5, FV+14 = 4/8/4, FV+28 = 4/6/5. (E, F) M/D: Day 0 = 5/1-4, FV+7 = 4-5/3-4, FV+14 = 4/6, FV+28 = 4/4. PA-D vaccinees returning in the PA-DB group are connected by lines. Bars represent medians. * *p* < 0.05, ** *p* < 0.01.

Using fluorophore-conjugated DBPRII protein probes to detect DBPRII-specific cells, we evaluated the capacity of the viral vector and protein/adjuvant platforms to drive a range of B cell responses (gating strategy shown in **Supplementary Figure 1A**). Within the plasma cell population (CD19+CD27+CD38+; **Figure 1A**) – which peaked 7 days after final vaccination – significantly higher frequencies of DBPRII-specific cells were observed in protein/adjuvant vaccinees compared to heterologous viral vector vaccinees at FV+7 and FV+14. Within the memory IgG+ B cell population (CD19+CD27+IgG+ [excluding plasma cells]; **Figure 1B**), frequencies of DBPRII-specific cells were significantly higher in protein/adjuvant vaccinees at all post-vaccination time points. Significant post-vaccination responses were observed within both of these B cell populations with both platforms (intra-trial statistics not shown). Consistent with expected differences in kinetics of short-lived plasma cell (SLPC) and memory B cell responses, DBPRII-specific plasma cells peaked earlier, at FV+7, and then declined by FV+28, whilst frequencies of DBPRII-specific memory IgG+ B cells were better maintained in peripheral blood between FV+7 and FV+28 [with a trend towards a peak at FV+14 (5)].

We next compared DBPRII-responses between the dosing regimens of the protein/adjuvant platform. Here, we observed more robust plasma cell (**Figure 1C**) and memory IgG+ B cell (**Figure 1D**) responses following delayed booster dosing (PA-D) compared to monthly booster dosing (PA-M). Proliferation – as indicated by intracellular Ki67 staining – was higher in plasma cells than memory B cells across all groups and time points (**Supplementary Figures 1B, C**). Differences between platforms and dosing regimens remained when we stratified between activated (CD21-CD27+) and resting (CD21+CD27+) memory IgG+ B cell populations (**Supplementary Figures 2A–D**). Interestingly, Ki67 expression was significantly higher in activated memory IgG+ B cells in PA-D at FV+7 compared to PA-M or PA-DB, and at FV+14 compared to PA-M (**Supplementary Figures 1C, D**). Very low memory IgA+ B cell responses were detectable (higher in protein/adjuvant than viral vector vaccinees), while responses within the IgM+ memory compartment were negligible (**Supplementary Figures 2E–H**).

We sought to validate these protein/adjuvant delayed booster dosing-mediated differences with samples from an independent clinical trial with a different cohort (Tanzanian adults) and antigen (RH5). Here, we observed comparable plasma cell and memory IgG+ B cell kinetics and differences in proliferation (**Figures 1E, F**; **Supplementary Figure 3**). Frequencies of RH5-specific cells within both populations were again higher in delayed booster dosing vaccinees but only reached statistical significance (by Mann Whitney test) for RH5-specific memory B cells at FV+14, likely related to greater intra-group variation, compared to the DBPRII vaccinees. Trends were comparable but not significant when activated and resting memory IgG+ B cells were analysed separately (**Supplementary Figure 4**). To note, while background with the RH5 probes was more variable than observed with the DBPRII probes, or as compared to previous studies with the RH5 probe protocol (5, 12), standardisation of RH5 probe gating between all samples and parent populations retains confidence in the data interpretation. No significant RH5-specific post-vaccination responses were observed for either the monthly or delayed boosting groups within the memory IgA+ or IgM+ populations, but further investigation may be of interest as trends appear different to memory IgG+ B cells (**Supplementary Figure 4**).

### Agnostically-defined B cell subsets reveal further differences in DBPRII- and RH5-specific B cell responses between vaccine platforms and dosing regimens

**Figure 2:**
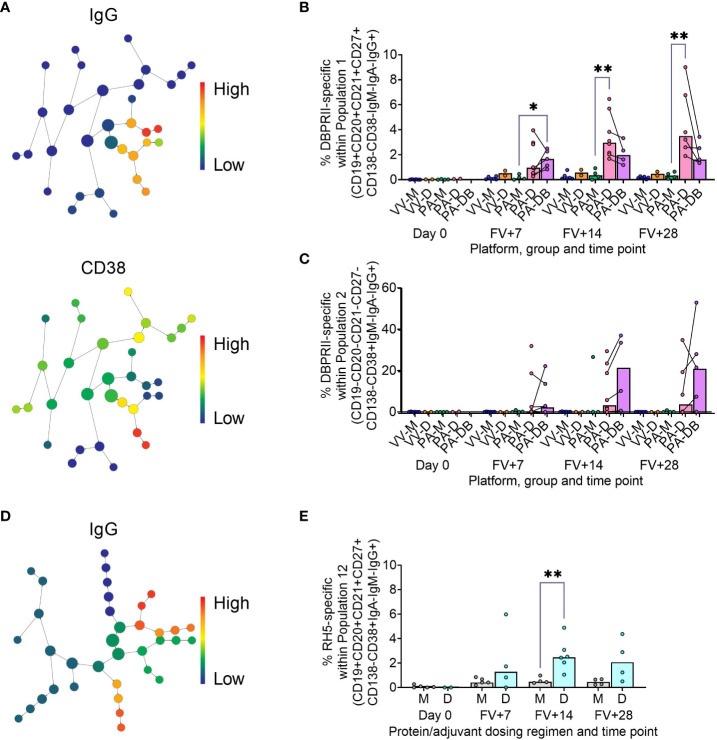
Vaccine-specific responses within agnostically-defined B cell populations using CITRUS. CITRUS was run on single live (B cell-enriched) lymphocyte flow cytometry fcs files to agnostically define the main B cell populations within either DBPRII **(A–C)** or RH5 **(D, E)** trial samples. Clusters identified by CITRUS are visualised in dendrograms **(A, D)**, colour-coded for example markers of interest [**(A)**- IgG, CD38; **(D)**- IgG]. Each node represents a cluster. Median marker expression within each cluster was used to define gating strategies for B cell populations in FlowJo, which were re-analysed for DBPRII- **(B, C)** or RH5-specific **(E)** responses through probe staining (gating shown in **Supplementary Figure 5**). See **Table 2** and **Supplementary Figures 6, 7** for a full list of populations identified via CITRUS clusters for further analysis. VV-M = ChAd63-MVA viral vector monthly dosing; VV-D ChAd63-MVA delayed booster dosing; PA-M = PvDBPII protein/adjuvant monthly dosing; PA-D = PvDBPII protein/adjuvant delayed booster dosing; PA-DB = PvDBPII protein/adjuvant delayed booster dosing with extra booster; M = RH5.1/adjuvant monthly dosing; D = RH5.1/adjuvant delayed booster dosing. FV = final vaccination. Post-vaccination comparisons were performed between PvDBPII protein/adjuvant dosing regimens by Kruskal Wallis test with Dunn’s correction for multiple comparisons **(B, C)** or RH5 dosing regimens **(E)** with Mann-Whitney U tests. Sample sizes for all assays were based on sample availability; each circle represents a single sample. **(B, C)** VV-M/VV-D/PA-M/PA-D/PA-DB: Day 0 = 6/2/4/2/na, FV+7 = 6/2/4/8/5, FV+14 = 6/2/4/8/4, FV+28 = 6/2/4/6/5. **(E)** M/D: Day 0 = 5/2, FV+7 = 5/4, FV+14 = 4/6, FV+28 = 4/4. PA-D vaccinees returning in the PA-DB group are connected by lines. Bars represent medians. * *p* < 0.05, ** *p* < 0.01.

To supplement the more traditional approach above, clustering was next performed with CITRUS to agnostically define B cell populations for further analysis within both the DBPRII and RH5 datasets. For the DBPRII trial samples, 33 clusters were identified in CITRUS within live single (B cell-enriched) lymphocytes. Several sets of clusters had very similar marker expression patterns, resulting in consolidation for FlowJo gating strategies (see Methods). This gave a total of 7 agnostically-defined populations to reanalyse for DBPRII-specific responses (**Table 2**). Likewise, 36 clusters were identified in the RH5 trial samples, consolidating to 10 new populations for reanalysis of RH5-specific responses (**Table 2**). Populations where significant differences in antigen-specific responses were detected post-vaccination or between dosing regimens are shown in **Figure 2** (gating shown in **Supplementary Figure 5**). DBPRII “Population 2” and RH5 “Population 8” were equivalent, while all other populations identified were unique to the separate trials.

**Table 2:** Main peripheral B cell populations as agnostically defined using CITRUS.

Trial	Population	FlowJo gating strategy						
		CD19	CD20	CD21	CD27	CD138	CD38	Isotype
DBPRII	1*	+	+	+	+	–	–	IgG
	2*^%^	–	–	–	–	–	+	IgG
	3	+	+	+	–	–	–	IgM
	4	–	–	–	–	–	–	IgG
	5	+	+	+	+	–	–	IgA
	6	+	+	+	–	+	–	IgM
	7	+	+	+	+	–	–	IgM
RH5	8^%^	–	–	–	–	–	+	IgG
	9	–	–	–	+	–	+	IgG
	10	+	+	+	–	–	+	IgM
	11	+	+	+	+	–	+	IgM
	12*	+	+	+	+	–	+	IgG
	13	+	+	+	+	–	+	IgA
	14	+	+	+	–	+	+	IgM
	15	–	–	–	–	–	–	–
	16	+	+	+	–	–	+	IgA
	17	+	+	+	–	–	+	–

NB population ordering and numbers are arbitrary.

* Significant antigen-specific responses detected within groups; shown in **Figure 2**; gating strategies shown in **Supplementary Figure 5**. ^%^ Equivalent populations identified independently.

Shading is to aid distinction between + and - cells.

In the DBPRII trial clusters, significant differences were observed between monthly and delayed protein/adjuvant dosing in “Population 1” (CD19+CD20+CD21+CD27+CD138-CD38-IgM-IgA-IgG+; **Figure 2B**; **Supplementary Figure 5**) and trends between monthly and delayed or delayed/booster (PA-DB) dosing in “Population 2” (CD19-CD20-CD21-CD27-CD138-CD38+IgM-IgA-IgG+; **Figure 2C**; **Supplementary Figure 5**). While “Population 1” is a subset of the CD19+CD21+CD27+IgG+ resting memory population analysed above (**Supplementary Figure 2**), “Population 2” was not included in the previous analysis. Responses within other clusters and frequencies of each cluster within total live (B cell-enriched) lymphocytes are shown in **Supplementary Figure 6**.

In the new RH5 trial populations, significant responses were observed in “Population 12” (**Figure 2E**; **Supplementary Figure 5**; CD19+CD20+CD21+CD27+CD138-CD38+IgM-IgA-IgG+; again, a subset of the CD19+CD21+CD27+IgG+ resting memory in **Supplementary Figure 4**) in both monthly and delayed booster protein/adjuvant dosing, with higher responses in delayed boosting vaccinees. Significant differences in post-vaccination RH5-specific responses were not detected between groups in the remaining populations (**Supplementary Figure 7**). No significant responses were observed within “Population 8” (CD19-CD20-CD21-CD27-CD138-CD38+IgM-IgA-IgG+; equivalent “Population 2” in the DBPRII trial) in the delayed boosting vaccinees.

### DBPRII-specific serum antibody declines more slowly in delayed booster dosing vaccinees

**Figure 3:**
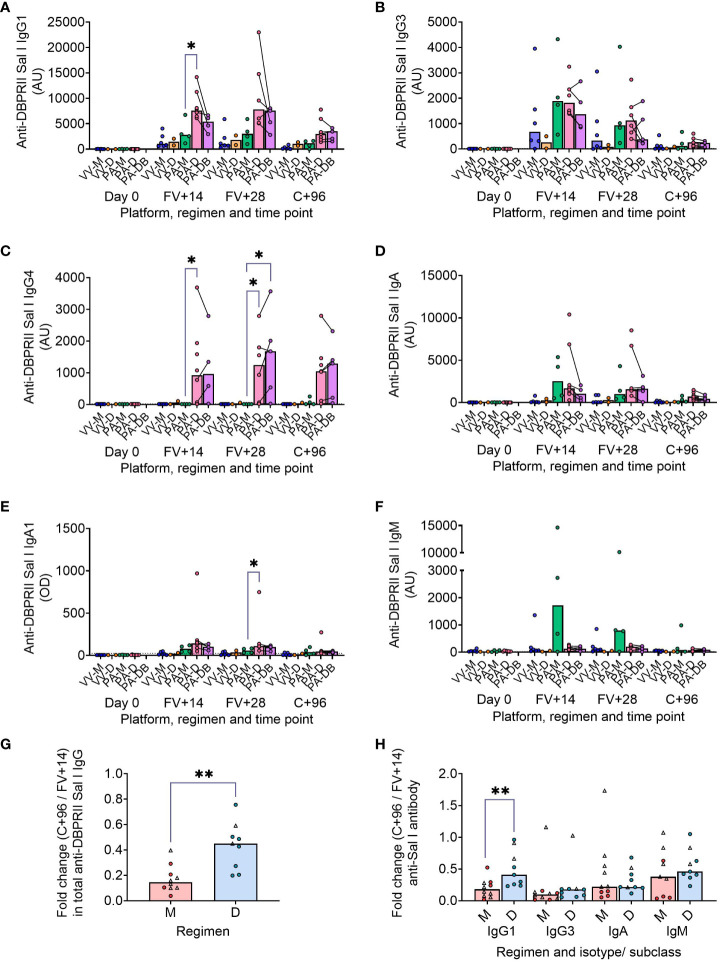
DBPRII-specific peak antibody responses and serum maintenance. Standardised ELISAs were developed to report anti-DBPRII specific antibody responses against the Sal I strain in pre-vaccination (Day 0) and post-final vaccination (FV) serum samples. Responses were compared between protein/adjuvant dosing regimens for IgG1 **(A)**, IgG3 **(B)**, IgG4 **(C)**, IgA **(D)**, IgA1 **(E)**, and IgM **(F)**. Fold change between C+96 and FV+14 was calculated for total IgG **(G)** and specific isotypes/subclasses **(H)** to compare monthly (M: VV-M, PA-M) and delayed (D: VV-D, PA-D) booster regimens. IgG4 and IgA1 were excluded from this analysis as ≥1 vaccinee had undetectable antibodies at both time points. Comparisons between vaccine platforms are shown in **Supplementary Figure 8**. VV-M = ChAd63-MVA viral vector monthly dosing; VV-D ChAd63-MVA delayed booster dosing; PA-M = protein/adjuvant monthly dosing; PA-D = protein/adjuvant delayed booster dosing; PA-DB = protein/adjuvant delayed booster dosing with extra booster. C+96 = 96 days after controlled human malaria infection (approximately 16 weeks after FV). Post-vaccination comparisons were performed between protein/adjuvant dosing regimens by Kruskal Wallis test with Dunn’s correction for multiple comparisons **(A–F)** or fold changes with Mann-Whitney U tests **(G, H)**. Sample sizes for all assays were based on sample availability; each circle represents a single sample [triangles indicate viral vector samples in **(G, H)**]. **(A–F)** VV-M/VV-D/PA-M/PA-D/PA-DB: Day 0 = 6/2/4/8/na, FV+14 = 6/2/4/8/4, FV+28 = 6/2/4/6/5. **(G, H)** M = 9-10, D = 9. Bars represent medians. * *p* < 0.05, ** *p* < 0.01.

We have previously published ELISA data on serum anti-DBPII total IgG (against the Sal I strain), with an emphasis on comparison of FV+14 responses. There, we observed significantly higher titres with a delayed protein/adjuvant dosing regimen compared to viral vectors (11). Now, we extend these analyses to compare the impact of platform and regimen on different isotypes/subclasses, (**Figures 3A–F**; **Supplementary Figure 8**), durability of serum antibody (**Figures 3G, H**), and immunodominance of subdomain 3 (sd3; **Supplementary Figure 8**). The isotype and subclass analyses showed IgG1, IgG3, IgA, and IgA1 responses in both platforms, while IgG4 was detectable solely in the protein/adjuvant vaccinees. No statistically significant post-vaccination IgM responses were observed within individual groups (**Supplementary Figure 8**), while no detectable IgG2 or IgA2 was observed in any sample (not shown). The protein/adjuvant platform also induced higher IgG1, IgG3, IgA, and IgA1 (and IgG4) responses compared to viral vectors (**Supplementary Figures 8A–F**). Within the protein/adjuvant platform, the DBPRII-specific IgG1, IgG4, and IgA1 response was significantly higher in delayed dosing vaccinees (**Figures 3A, C, E**) but comparable between regimens for IgG3 and IgA (**Figures 3B, D**). Median IgM responses were higher in monthly dosing but not statistically significant (**Figure 3F**). Interestingly, we also observed increased anti-DBPRII serum total IgG durability in delayed dosing regimens with both vaccine platforms; a significantly higher fold-change in total serum IgG was observed > 3 months following the peak response in delayed booster vaccinees (VV-D, PA-D; median = 0.45) compared to monthly dosing vaccinees (VV-M, PA-M; median = 0.15; **Figure 3G**). Enhanced serum durability over > 3 months was also observed with IgG1 in the isotype and subclass analyses (**Figure 3H**). Analyses with even later serum time points (e.g., 6 months or 1 year after final vaccination) were not feasible in the current study but would be of interest to understand longer-term durability. Finally, delayed dosing with PvDBPII appears to have no impact on the immunodominance of the sd3 region (**Supplementary Figure 8**). Equivalent serological analyses from the RH5.1/Matrix-M™ trial will be the focus of a separate report (Silk et al., *manuscript in preparation*).

### Delayed booster dosing does not impact DBPRII-specific T-cell responses

**Figure 4:**
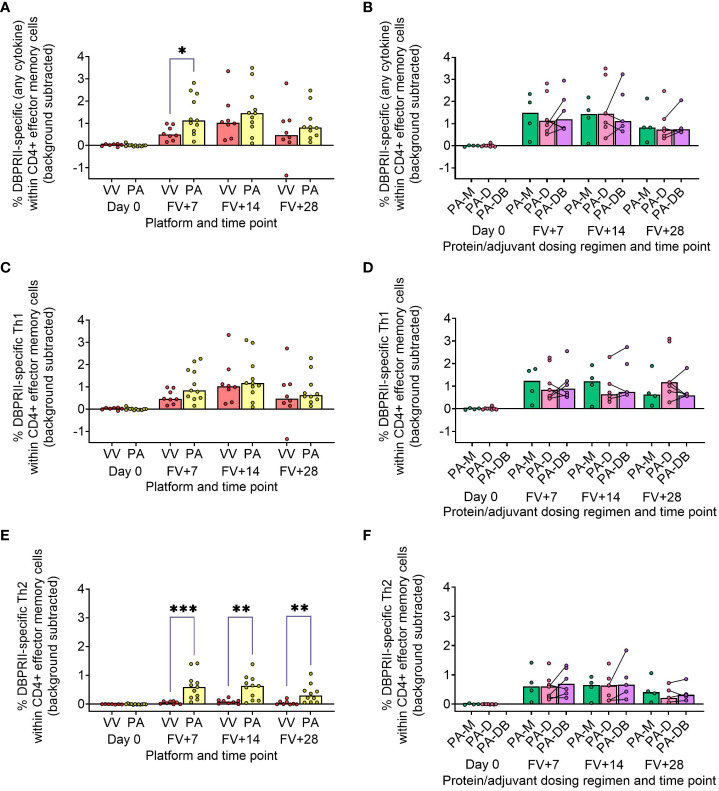
DBPRII-specific CD4+ effector memory T cell responses. PBMC from pre-vaccination (Day 0) and post-final vaccination (FV) time points were analysed for T cell responses by intracellular cytokine staining; gating strategies are as described in Methods and **Supplementary Figure 9**. In brief, DBPRII-specific effector memory CD4+ T cells are reported as frequencies producing cytokines in response to peptide stimulation after background subtraction of cytokine-positive cells in matched samples cultured with media alone. DBPRII-specific responses were compared between vaccine platforms or protein/adjuvant dosing regimens as defined by the production of any cytokine [IL-2, IL-5, IL-13, IFN-γ, TNF-α] **(A, B)**, any Th1 cytokine [IL-2, IFN-γ, TNF-α] **(C, D)**, or any Th2 cytokine [IL-5, IL-13] **(E, F)**. CD8+ effector memory T-cell responses are shown in **Supplementary Figure 10**. VV = ChAd63-MVA viral vectors [monthly and delayed dosing]; PA = PvDBPII protein/adjuvant [PA-M and PA-D]; PA-M = PvDBPII protein/adjuvant monthly dosing; PA-D = PvDBPII protein/adjuvant delayed booster dosing; PA-DB = PvDBPII protein/adjuvant delayed booster dosing with an extra booster. Post-vaccination comparisons were performed between PvDBPII platforms by Mann Whitney U test **(A, C, E)**, or protein/adjuvant dosing regimens by Kruskal Wallis test with Dunn’s correction for multiple comparisons **(B, D, F)**. Sample sizes for all assays were based on sample availability; each circle represents a single sample. **(A, C, E)** VV/PA: Day 0 = 7/12, FV+7 = 8/11, FV+14 = 8/11, FV+28 = 8/10. **(B, D, E)** PA-M/PA-D/PA-DB: Day 0 = 4/8/na, FV+7 = 4/7/6, FV+14 = 4/7/5, FV+28 = 4/6/5. PA-D vaccinees returning in the PA-DB group are connected by lines. Bars represent medians. * *p* < 0.05, ** *p* < 0.01, *** *p* < 0.001.

In our main trial report, we showed higher frequencies of IFN-γ-producing effector memory (CD45RA-CCR7-) CD4+ T cells at FV+14 in viral vectors compared to protein/adjuvant vaccinees, with no significant differences observed between monthly and delayed dosing regimens with the latter platform (11). Here, we extended these analyses to include further time points as well as IL-2/TNF-α/IL-5/IL-13 intracellular cytokine detection (in addition to IFN-γ), allowing a more nuanced comparison of responses between vaccine platform and regimen. Looking first at all DBPRII-specific effector memory CD4+ T cells – based on the secretion of any cytokine following stimulation with the DBPRII peptide pool (**Table 3**) – we observed significantly higher frequencies in protein/adjuvant vaccinees at FV+7 compared to viral vector vaccinees but no difference between dosing regimens (**Figures 4A, B**). Moving from this measure of the magnitude of the DBPRII-specific CD4+ T cell response to more qualitative read-outs, within the same effector memory CD4+ T cell population, we next assessed total Th1 (defined as IFN-γ and/or IL-2 and/or TNF-α) and Th2 responses (IL-5 and/or IL-13). Both platforms induced Th1 cytokine production and while there were no significant differences between platforms in the total Th1 response (**Figure 4C**), viral vector vaccinees trended towards higher IFN-γ production versus higher IL-2 with protein/adjuvant vaccinees (**Supplementary Figure 9**). No significant differences between protein/adjuvant dosing regimens were detected when Th1 cytokines were analysed together (**Figure 4D**) or individually (data not shown). In contrast, the protein/adjuvant vaccines drove higher frequency total Th2 responses, comparable across different dosing regimens (**Figures 4E, F**). Trends were similar for both IL-5 and IL-13, although the magnitude of DBPRII-specific IL-5-producing cells was higher (**Supplementary Figure 9**).

**Table 3:** Peptide pool for T cell stimulation.

Peptide Number	N-terminus	Amino Acid Sequence	C-terminus
1	H-	DHKKTISSAIINHAFLQNTVGSG(261)	-NH2
2	Biotin-	SGSGAIINHAFLQNTVMKNCNYKR	-NH2
3	Biotin-	SGSGQNTVMKNCNYKRKRRERDWD	-NH2
4	Biotin-	SGSGNYKRKRRERDWDCNTKKDVC	-NH2
5	Biotin-	SGSGRDWDCNTKKDVCIPDRRYQL	-NH2
6	Biotin-	SGSGKDVCIPDRRYQLCMKELTNL	-NH2
7	Biotin-	SGSGRYQLCMKELTNLVNNTDTNF	-NH2
8	Biotin-	SGSGLTNLVNNTDTNFHRDITFRK	-NH2
9	Biotin-	SGSGDTNFHRDITFRKLYLKRKLI	-NH2
10	Biotin-	SGSGTFRKLYLKRKLIYDAAVEGD	-NH2
11	Biotin-	SGSGRKLIYDAAVEGDLLLKLNNY	-NH2
12	Biotin-	SGSGVEGDLLLKLNNYRYNKDFCK	-NH2
13	Biotin-	SGSGLNNYRYNKDFCKDIRWSLGD	-NH2
14	Biotin-	SGSGDFCKDIRWSLGDFGDIIMGT	-NH2
15	Biotin-	SGSGSLGDFGDIIMGTDMEGIGYS	-NH2
16	Biotin-	SGSGIMGTDMEGIGYSKVVENNLR	-NH2
17	Biotin-	SGSGIGYSKVVENNLRSIFGTDEK	-NH2
18	Biotin-	SGSGNNLRSIFGTDEKAQQRRKQW	-NH2
19	Biotin-	SGSGTDEKAQQRRKQWWNESKAQI	-NH2
20	Biotin-	SGSGRKQWWNESKAQIWTAMMYSV	-NH2
21	Biotin-	SGSGKAQIWTAMMYSVKKRLKGNF	-NH2
22	Biotin-	SGSGMYSVKKRLKGNFIWICKLNV	-NH2
23	Biotin-	SGSGKGNFIWICKLNVAVNIEPQI	-NH2
24	Biotin-	SGSGKLNVAVNIEPQIYRWIREWG	-NH2
25	Biotin-	SGSGEPQIYRWIREWGRDYVSELP	-NH2
26	Biotin-	SGSGREWGRDYVSELPTEVQKLKE	-NH2
27	Biotin-	SGSGSELPTEVQKLKEKCDGKINY	-NH2
28	Biotin-	SGSGKLKEKCDGKINYTDKKVCKV	-NH2
29	Biotin-	SGSGKINYTDKKVCKVPPCQNACK	-NH2
30	Biotin-	SGSGVCKVPPCQNACKSYDQWITR	-NH2
31	Biotin-	SGSGNACKSYDQWITRKKNQWDVL	-NH2
32	Biotin-	SGSGWITRKKNQWDVLSNKFISVK	-NH2
33	Biotin-	SGSGWDVLSNKFISVKNAEKVQTA	-NH2
34	Biotin-	SGSGISVKNAEKVQTAGIVTPYDI	-NH2
35	Biotin-	SGSGVQTAGIVTPYDILKQELDEF	-NH2
36	Biotin-	SGSGPYDILKQELDEFNEVAFENE	-NH2
37	Biotin-	SGSGLDEFNEVAFENEINKRDGAY	-NH2
38	Biotin-	SGSGFENEINKRDGAYIELCVCSV	-NH2
39	Biotin-	SGSGDGAYIELCVCSVEEAKKNTQ	-NH2
40	Biotin-	SGSGIELCVCSVEEAKKNTQEVVT	-OH

The PvDBPII SalI amino acid sequence was used to design 20mer peptides overlapping by 12 amino acids and these were synthesised by Mimotopes, Australia. Each stock was reconstituted to 50mg/mL in DMSO. A 200µg/peptide/mL working stock of PvDBPII peptides was prepared by adding an equal amount of each peptide to cell culture medium for a final total peptide concentration of 8mg/mL.

Finally, we similarly quantified the effector memory CD8+ T cell response. Here, Th1 responses were observed only within the viral vector platform, whether by total Th1 or by individual cytokine (**Supplementary Figure 10**).

### DBPRII-specific B cell responses correlate with *in vivo* efficacy against *P. vivax* parasites and durability of anti-DBPRII serum IgG

**Figure 5:**
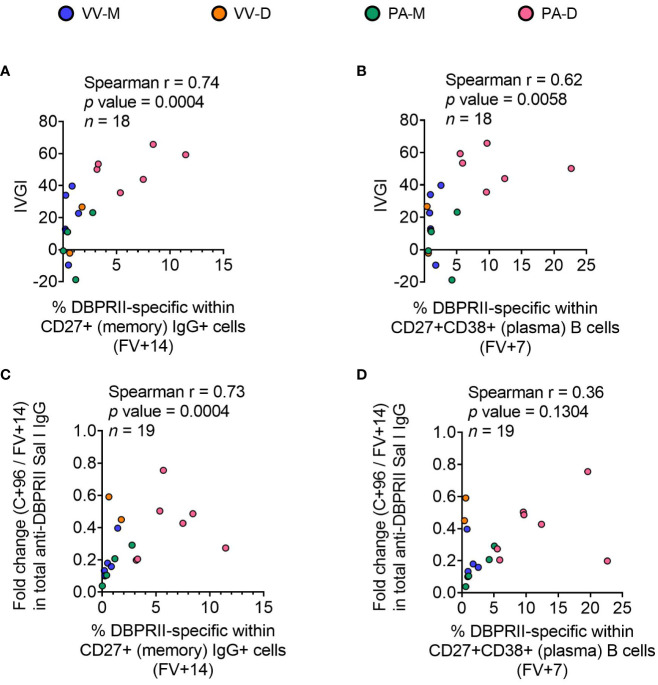
Correlations between circulating DBPRII-specific B cells and *in vivo* growth inhibition of *P. vivax* parasites or maintenance of serum antibody. *In vivo* growth inhibition (IVGI) of *P. vivax* parasites following post-vaccination controlled human malaria infection (CHMI) was calculated from qPCR data as described in the Methods. Spearman correlations were performed between IVGI and the peak frequency of DBPRII-specific memory IgG+ B cells at FV+14 **(A)** or plasma cells at FV+7 **(B)** as defined in **Supplementary Figure 1** and reported in **Figure 1**. Spearman correlations were also performed between C+96/FV+14 fold change in total anti-DBPRII IgG (Sal I strain; see **Figure 3**) and memory IgG+ B cells at FV+14 **(C)** or plasma cell at FV+7 **(D)**. VV-M = ChAd63-MVA viral vector monthly dosing; VV-D ChAd63-MVA delayed booster dosing; PA-M = protein/adjuvant monthly dosing; PA-D = protein/adjuvant delayed booster dosing. C+96 = 96 days after controlled human malaria infection (approximately 16 weeks after FV). Spearman rho, *p*-values, and sample sizes are annotated on individual graphs. Each circle represents a single sample.

To confirm the biological relevance of increased B cell immunogenicity in delayed dosing vaccinees, we performed Spearman correlations with *in vivo* growth inhibition of blood-stage parasites following *P. vivax* CHMI (comparisons of parasite growth dynamics by group have previously been reported (11)). The magnitude of the circulating DBPRII-specific memory IgG+ B cell response correlated strongly with IVGI (**Figure 5A**), as did the frequency of DBPRII-specific plasma cells (**Figure 5B**). Since IVGI is calculated over a relatively short time frame following parasite inoculation, we were also interested in assessing the relationship between these B cell responses and the fold change in serum antibody between the peak (FV+14) and a later time point (C+96, approximately 16-weeks later; **Figure 3G**). DBPRII-specific IgG+ memory B cells again correlated strongly with durable serum anti-DBPRII IgG responses (**Figure 5C**), while no association was observed with peak FV+7 plasma cells (**Figure 5D**). Neither IVGI nor serum antibody maintenance correlated with DBPRII-specific Th2 effector memory CD4+ T cell responses (data not shown), suggesting a lesser role for T cell immunogenicity in DBPRII vaccine-mediated protection.

### Bone marrow plasma cells correlate with serum antibody and circulating memory B cells in adult RH5.1/Matrix-M™ vaccinees

**Figure 6:**
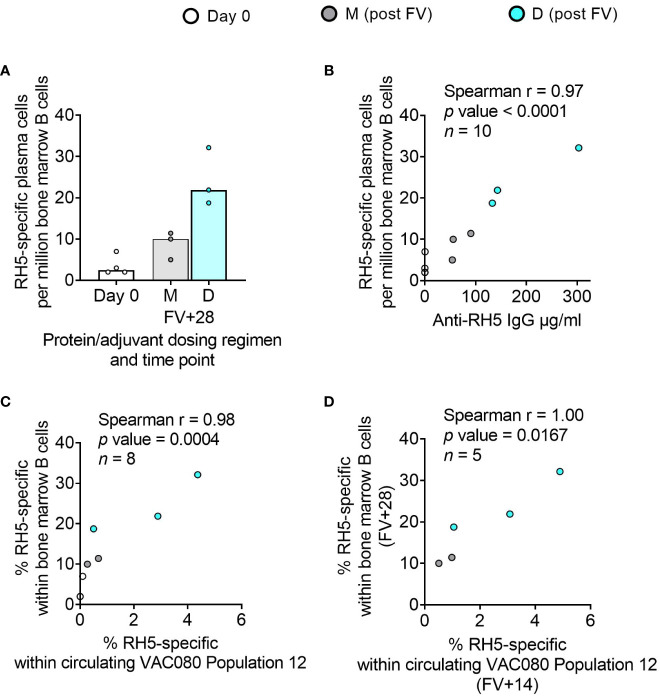
RH5-specific bone marrow plasma cell responses and correlations with serum antibody or circulating RH5-specific cells. RH5-specific bone marrow plasma cells were detected in B cells enriched from pre- and post-final vaccination (FV) bone marrow mononuclear cells and assayed by IgG antibody-secreting cell ELISPOT as described in the Methods. The frequency of RH5-specific IgG plasma cells (antibody-secreting cells) per million bone marrow B cells was compared between dosing regimens **(A)**. Spearman correlation analyses were performed between RH5-specific bone marrow B cells and matched time point serum IgG **(B)**, matched time point frequency of RH5-specific cells within CITRUS-guided “Population 12” [CD19+CD20+CD21+CD27+CD138-CD38+IgM-IgA-IgG+; see **Table 2**, **Figure 2**, **Supplementary Figure 5**] **(C)**, and between RH5-specific bone marrow B cells at FV+28 and “Population 12” at FV+14 **(D)**. M = RH5.1/adjuvant monthly dosing; D = RH5.1/adjuvant delayed booster dosing. Post-vaccination comparisons were performed between dosing regimens by Mann Whitney U test [**(A)**; not significant]. Spearman rho, p-values, and sample sizes are annotated on individual graphs. Each circle represents a single sample.

While bone marrow aspirates were not taken in the DBPRII clinical trials, we had the rare opportunity to investigate bone marrow plasma cells with the RH5.1/Matrix-M™ adult vaccinees – the first bone marrow analyses in the context of malaria vaccination (13). Here, we observed a trend to higher frequencies of RH5-specific cells within total bone marrow B cells in the protein/adjuvant delayed dosing regimen (**Figure 6A**), which correlated strongly with matched time point serum anti-RH5 IgG (**Figure 6B**). We were also interested to see whether the frequencies of RH5-specific cells within circulating B cell populations of interest correlated with vaccine-specific seeding of the bone marrow. We therefore performed additional Spearman correlation analyses between the frequency of RH5-specific cells within “Population 12” (CD19+CD20+CD21+CD27+CD138-CD38+IgA-IgM-IgG+; the B cell population with greatest differences between regimens) at both matched time points (i.e., V1 or FV+28; **Figure 6C**) or with FV+14 (**Figure 6D**). We observed strong correlations between RH5-specific circulating memory B cells and bone marrow plasma cells at both time points. Similar results were obtained if correlations were performed with RH5-specific CD19+CD27+IgG+ B cells (**Figure 1F**; data not shown). RH5-specific bone marrow B cells did not correlate with circulating RH5-specific CD27+CD38+ (plasma) cells at FV+7 (data not shown).

## Discussion

The exploratory data presented here contribute to growing efforts seeking to understand the impact of modifiable vaccine delivery parameters – i.e. vaccine platform or dosing regimen – on protective immune responses. Using samples from independent clinical trials with the blood-stage malaria antigens DBPRII and RH5, we observe a hierarchy of serum antibody and B cell immunogenicity from heterologous viral vectors to monthly protein/adjuvant booster dosing to delayed booster protein/adjuvant vaccination. Consistent with a role for vaccine-specific B cells in sustained humoral immunity, circulating plasma or memory B cell responses correlate with available *in vivo* growth inhibition (IVGI) and durable serum IgG data from the DBPRII trial. In fact, DBPRII-specific memory IgG+ B cells correlate more strongly with IVGI than our previously reported associations between IVGI and serum anti-DBPRII IgG, DARC (the DBP ligand) binding inhibition, or *in vitro* growth inhibitory activity (GIA) with a transgenic *P. knowlesi* strain expressing *P. vivax* DBPRII (11). In contrast, while differences in CD4+ and CD8+ T cell responses are observed between platforms by intracellular cytokine staining (ICS), there are no discernible differences between protein/adjuvant dosing regimens and no correlation is observed between IVGI and T cell immunogenicity (11).

Our conclusions are in line with data published by Payne et al., showing an increase in SARS-CoV-2 spike-specific IgG+ antibody-secreting B cells by ELISPOT 4 weeks after the second dose with longer intervals between BNT162b2 mRNA doses in naïve vaccinees (median = 3.4 weeks, versus median = 10.1 weeks). More mixed results were observed with T cell immunogenicity. For example, the authors observed decreased spike-specific IFN-γ ELISPOT responses and CD8+ IFN-γ by ICS, alongside increased spike-specific CD4+ IL-2 and IFN-γ by ICS (3). Likewise, recent work from Nicolas et al. reported “long interval” (i.e., delayed) booster dosing increases circulating SARS-CoV-2 RBD-specific IgG+ B cells 1-3 weeks after the second mRNA dose, without driving major differences in memory CD4+ or CD8+ T cells [as measured by activation-induced marker [AIM] or ICS (14)]. Our own previously published work with a similar AIM assay in the context of a UK RH5.1/AS01 Phase I vaccine trial showed no effect of delayed boosting on the magnitude of the Tfh cell response, but we did detect a slight shift towards a Tfh2 phenotype as compared to monthly boosting vaccinees (4). To note, the Nicolas et al. study compared “short” (median = 3.0 weeks) and “long” (median = 15.8 weeks) intervals between the two mRNA doses similar to the spacing between 2^nd^ and 3^rd^ doses in the RH5 and CSP (RTS,S) trials. However, for many comparisons of parameters between the malaria and SARS-CoV-2 fields, it is important to remember that the “delay” is often less substantial in SARS-CoV-2 trials [i.e., a few weeks (1–3), rather than several months, as tested with DBPRII/RH5/CSP- based vaccines (4–9, 11)]. We are far from understanding the optimal spacing of booster doses, but if the benefit to B cell immunogenicity with delayed booster vaccination relates to allowing circulating antibody and/or ongoing germinal centres to wane, as we have previously proposed (5), then it seems likely that this difference of weeks versus months will be immunologically relevant.

Our approach to the B cell flow cytometry analyses also incorporated an agnostic approach to identifying the main circulating B cell populations through the use of the CITRUS clustering tool. Here, the goal was to complement, not replace, the more traditional pre-defined flow cytometry gating strategies with a more holistic approach. Significant post-vaccination responses were observed within DBPRII “Population 1” and RH5 “Population 12”, which appear to be subsets of the resting memory populations. Further interrogation of these populations to understand functional differences to other memory IgG+ B cell populations – and the significance of CD38 expression – would be of value. Interestingly, within the DBPRII trial, substantial post-vaccination responses within the protein/adjuvant delayed boosting groups were detected in a new “Population 2” (CD19-CD20-CD21-CD27-CD138-CD38+IgM-IgA-IgG+). This was an unexpected observation that would have been missed with traditional B cell analytical approaches that start from the premise that all B cells are CD19+. Given that our flow cytometry assay includes a negative pan B cell enrichment step and IgG expression is not expected on other lymphocytes, it is likely that the vast majority of these CD38+IgG+ cells are true B cells. At present, there appears to be limited and conflicting published data on similar (healthy) human vaccine-specific CD19- B cells in circulation. For example, Arumugakani et al. have reported on influenza-specific IgG-secreting CD19-CD20-CD38hi cells by ELISPOT and concluded this population was at the transition from plasmablast to mature plasma cell but, in contrast to our “Population 2” data this population was also CD27hi (15). Conversely, Mei et al. did not detect post-vaccination tetanus-toxoid-specific cells within circulating CD19-CD38+IgG+ B cells by intracellular probe staining and concluded plasma cell CD19 downregulation does not occur until *in situ* in the bone marrow (16). Whether “Population 2” overlaps with either of these published populations and whether it contains long-lived plasma cell precursors remain intriguing open questions.

In light of the interest in CD19- B cells in the context of long-lived plasma cell responses, future trials with larger sample sizes should include immunokinetic investigations of CD19- subpopulations and their biological significance. Indeed, of great interest is the strong correlation detected between circulating serum IgG or memory B cells and vaccine-specific bone marrow plasma cells in the RH5 trial. Very few vaccine studies have included lymphoid tissue sampling [reviewed in (13)] and, to the best of our knowledge, this trial represents the first direct analysis of human bone marrow-resident plasma cells in the context of malaria. Since long-term serum antibody is maintained through the secretion by long-lived plasma cells in the bone marrow, understanding the factors that impact this compartment is central to optimising the durability of humoral immunity. Our data strongly suggest that delayed protein/adjuvant dosing improves the generation of long-lived plasma cells in draining lymph node germinal centres and the subsequent seeding of bone marrow plasma cells as compared to monthly booster dosing. Future studies should build on these exciting findings with larger sample sizes or sample volumes, which would permit more detailed analyses of populations of interest, such as the putative long-lived plasma cell population [CD19-CD38+CD138+ (16–18)] within total bone marrow plasma cells. Similarly, the inclusion of draining lymph node fine needle aspirates would enable immunokinetic analyses of upstream germinal centre reactions. Given that our previously published work showed that delayed booster dosing drove increased RH5-specific serum IgG avidity and increased RH5-specific CDR3 percentage mutation from germline (4, 5), we would anticipate the detection of longer-lasting germinal centres with delayed booster dosing rather than monthly booster dosing. This would be consistent with both an improved long-lived plasma cell output and increased affinity maturation.

There are also several aspects of the DBPRII serology data that deserve further discussion. Firstly, it is interesting to note that median peak responses after an additional booster vaccination (PA-DB vs PA-D) are lower across the majority of isotypes and subclasses measured. This is mirrored in the DBPRII-specific B cell data and indeed reaches statistical significance for the CD27+CD38+ plasma cell response. The inclusion of germinal centre analyses in future work may help illuminate why PA-DB dosing did not boost responses beyond what was observed with the PA-D regimen. The exception is serum IgG4, which trends to a higher peak concentration following PA-DB compared to the PA-D regimen, and we have previously observed an enhanced IgG4 response with higher antigen doses of RH5.1/AS01 in an equivalent UK population (5). Secondly, we were surprised by the absence of detectable IgG2 following vaccination with any of the platforms/regimens. This is in contrast to the previous RH5 analyses, where we observed a (low) IgG2 response to both monthly and delayed booster dosing regimens, with better serum maintenance in the latter group (5). Finally, while intragroup variation and small sample sizes reduced the statistical power to detect differences in the IgM analyses, it is interesting to note that median responses were higher in the monthly dosing regimen compared to the delayed dosing regimen (FV+14: PA-M = 1708 AU, PA-D = 119.8 AU, PA-DB = 126.0 AU; FV+28: PA-M = 794.1 AU, PA-D = 204.4 AU, PA-DB = 129.4 AU). Although frequencies of DBPRII-specific B cells within the memory IgM+ population were very low, these did trend to slightly higher medians at FV+28 and correlate with serum IgM (Spearman r = 0.69, *p* = 0.0014, *n* = 18).

Given the multitude of parameters assayed, machine learning – such as with the SIMON platform (19) – would have been a useful strategy for identifying which read-out or set of read-outs best predicted our trial outcomes of interest, e.g., peak vaccine-specific IgG, IVGI (DBPRII trial only), or seeding of bone marrow plasma cells (RH5 trial only). Unfortunately, this approach was precluded by insufficient sample size. Indeed, the small sample sizes of the different vaccination groups represents the main limitation of our analyses for both trials.

To conclude, our exploratory data indicate that while changing the vaccine platform drives broad effects on post-vaccination immune responses, modulating booster dosing regimen more narrowly impacts humoral immunity. This is most evident for the protein/adjuvant platform where our clinical trial design and sample sizes enabled more detailed analyses. Importantly, the differences in B cell immunogenicity appear to have relevance for protection from *P. vivax* in a CHMI model. While this investigation of delayed boosting was a serendipitous effect of the SARS-CoV-2 pandemic – not unlike the original delayed fractional booster observations with RTS,S (6) – it now seems likely that future clinical development of the protein/adjuvant PvDBPII candidate will benefit from further interrogation of delayed booster regimens. These findings are supported by data from an independent RH5.1/Matrix-M™ clinical trial in malaria-exposed adults in Tanzania, whereby delayed protein/adjuvant booster dosing was found to not only increase the frequency of circulating RH5-specific memory B cells but also to increase RH5-specific plasma cells in the bone marrow.

## Methods

### Clinical trials

This study focused on the comparison of immune responses between groups vaccinated with the *Plasmodium vivax* antigen DBPRII with different platforms and dosing regimens [**Table 1**, (11)]. In brief, two Phase I/IIa vaccine efficacy trials (NCT04009096 and NCT04201431) were conducted in parallel at a single site in the UK (Centre for Clinical Vaccinology and Tropical Medicine, University of Oxford). NCT04009096 was an open-label trial to assess the ChAd63 and MVA viral-vectored vaccines encoding PvDBPII (VV-PvDBPII), while the NCT04201431 trial assessed the protein vaccine PvDBPII in Matrix-M™ adjuvant from Novavax (PvDBPII/M-M). NCT04009096 viral vectors were administered at 0 and 2 months (VV-M), or in a delayed dosing regimen, at 0, 17, and 19 months (VV-D). For NCT04201431, the protein/adjuvant was administered monthly (0, 1, 2 months; PA-M), or in a delayed dosing regimen, at 0, 1, 14 months (PA-D). A subset of vaccinees from the protein/adjuvant delayed dosing regimen returned for an additional booster at 19 months (PA-DB). ChAd63-PvDBPII was administered at a dose of 5×10^10^ viral particles, MVA-PvDBPII at 2×10^8^ plaque forming units, and PvDBPII protein at 50μg mixed with 50μg Matrix-M™. Delayed regimens were due to trial halts during the pandemic. Eligible vaccinees were healthy, Duffy-positive, malaria-naïve adults, aged 18 to 45 years. Trials were approved by the UK National Health Service Research Ethics Services (REC; references 19/SC/0193 and 19/SC/0330) and the UK Medicine and Healthcare products Regulatory Agency (MHRA; reference CTA 21584/0414/001-0001 and CTA 21584/0418/001-0001). All vaccinees gave written informed consent.

This study also includes analyses of samples from a further Phase Ib clinical trial with *Plasmodium falciparum* vaccine candidate RH5.1 (50µg) in Matrix-M™ adjuvant (50µg) in a malaria-endemic setting in Tanzania (NCT04318002). RH5.1/Matrix-M™ was administered either monthly, at 0, 1, and 2 months (M), or in a delayed booster regimen, at 0, 1, and 6 months (D). The final vaccination in the delayed booster regimen was given at a fractionated RH5.1 antigen dose of 10µg, rather than 50µg (the dose of Matrix-M™ was not fractionated and remained at 50 µg). Eligible vaccinees were healthy adults (negative for malaria by blood smear at screening), aged 18 to 45 years. The trial was approved by the Tanzanian Medicines and Medical Devices Authority (reference TMDA0020/CTR/0006/01), the National Institute for Medical Research (references NIMR/HQ/R.8a/Vol.IX/3537 and NIMR/HQ/R.8c/Vol.1/1887), the Ifakara Health Institute Institutional Review Board (reference IHI/IRB/No:49-2020), and the Oxford Tropical Research Ethics Committee (reference 9-20). All vaccinees gave written informed consent.

### Methods details

#### Flow cytometry – B cells

Cryopreserved PBMC from the DBPRII trial were thawed into R10 media (RPMI [R0883, Sigma] supplemented with 10% heat-inactivated FCS [60923, Biosera], 100U/ml penicillin/0.1mg/mL streptomycin [P0781, Sigma], and 2mM L-glutamine [G7513, Sigma]) then washed and rested in R10 for 1h. B cells were enriched (Human Pan-B cell Enrichment Kit [19554, StemCell]) and then stained with viability dye FVS780 (565388, BD Biosciences). Next, B cells were stained with anti-human CD19-BV786 (563325, BD Biosciences), anti-human CD20-BUV395 (563782, BD Biosciences), anti-human IgG-BB515 (564581, BD Biosciences), anti-human IgM-BV605 (562977, BD Biosciences), anti-human CD27-PE-Cy7 (560609, BD Biosciences), anti-human CD21-BV711 (563163, BD Biosciences), anti-human CD38-BV480 (566137, BD Biosciences), anti-human CD138-APC-R700 (566050, BD Biosciences), anti-human IgA-PerCP-Vio700 (130-113-478, Miltenyi), and two fluorophore-conjugated DBPRII probes. Preparation of the DBPRII probes was based on our previously published protocols with the *P. falciparum* blood-stage malaria antigen RH5 (5, 12). In brief, monobiotinylated DBPRII was produced by transient co-transfection of HEK293F cells with a plasmid encoding BirA biotin ligase and a plasmid encoding a monoFC-fused, biotin acceptor peptide- and c-tagged full-length DBPRII. Monobiotinylated DBPRII was purified by affinity chromatography (c-tag) and size exclusion chromatography. The monoFC solubilisation domain was cleaved using TEV protease. Probes were freshly prepared for each experiment by incubation of monobiotinylated DBPRII with streptavidin-PE (S866, Invitrogen) or streptavidin-APC (Biolegend, 17-4317-82) at a molar ratio of approximately 4:1to facilitate tetramer generation and, subsequently, centrifuged to remove aggregates. Following surface staining, cells were permeabilised and fixed with Transcription Factor Buffer Set (562574, BD Biosciences), stained with anti-human Ki67-BV650 (563757, BD Biosciences), washed, and stored at 4°C until acquisition. Samples were acquired on a Fortessa X20 flow cytometer with FACSDiva8.0 (both BD Biosciences). Samples were analysed using FlowJo (v10; Treestar). Samples were excluded from analysis if there were < 50 cells in the parent population.

The B cell assay with cryopreserved samples from the RH5 clinical trial was performed as above with two modifications to the protocol. First, RH5 probes rather than DBPRII probes were used as previously described (5, 12). Second, probe staining was repeated during the intracellular cytokine staining step at a 1/10 dilution as compared to concentrations used for surface staining.

See below for details of CITRUS analyses with B cell flow cytometry samples.

#### Flow cytometry – T cells

DBPRII peptide stimulation was used to detect DBPRII-specific T cells in an intracellular cytokine staining (ICS) assay as previously described (11). Cryopreserved PBMC were thawed in R10 and rested before an 18h stimulation with medium alone, 2.5 µg/peptide/mL of a PvDBPII 20mer peptide pool (Mimotopes; **Table 3**), or 1 µg/mL Staphylococcal enterotoxin B (SEB; S-4881, Sigma; positive control). Anti-CD28 (1µg/ml; 16-0289-85, eBioscience), anti-CD49d (1µg/ml; 16-0499-85, eBioscience), and anti-CD107a-PE-Cy5 (15-1079-42, eBioscience) were included in the cell culture medium. Brefeldin A (00-4506-51, eBioscience) and monensin (00-4505-51, eBioscience) were added after 2h. Following incubation, PBMC were stained with viability dye Live/Dead Aqua (L34966, Invitrogen) and anti-human CCR7-BV711 (353228, Biolegend). Cells were then permeabilised and fixed with Cytofix/Cytoperm (554714, BD Biosciences) before staining with anti-human CD14-eF450 (48-0149-42, eBioscience), anti-human CD19-eFl450 (48-0199-42, eBioscience), anti-human CD8a-APC-eF780 (47-0088-42, eBioscience), anti-human IFN-γ-FITC (11-7319-82, eBioscience), anti-human TNFα-PE-Cy7 (25-7349-8, eBioscience), anti-human CD3-AF700 (56-0038-82, eBioscience), anti-CD4-PerCP Cy5.5 (300530, Biolegend), anti-human IL-2-BV650 (500334, Biolegend), anti-human IL5-PE (500904, Biolegend), anti-human IL13-APC (501907 Biolegend), anti-human CD45RA-BV605 (304134, Biolegend). Finally, cells were washed, and stored at 4°C until acquisition. Samples were acquired on a Fortessa X20 flow cytometer with FACSDiva8.0 (both BD Biosciences). Samples were analysed using FlowJo (v10; Treestar). Background cytokine responses to medium alone were subtracted from DBPRII-specific responses. Samples were excluded from analysis if there were <50 cells in the parent population.

### ELISAs

For the DBPRII clinical trial, antigen-specific total IgG, IgG3, IgG4, IgA, IgA1, and IgM titres were determined by standardised ELISA in accordance with published methodology (20). Nunc MaxiSorp™ flat-bottom ELISA plates (44-2404-21, Invitrogen) were coated overnight with 2µg/mL (for total IgG titres) or 5µg/mL (for IgG1, IgG3, IgG4, IgA, IgA1, and IgM titres) of DBPRII SalI protein or 2µg/mL of sd3 protein in PBS. DBPRII protein was produced as previously described (11), while sd3 protein was produced by transient transfection of Expi395F cells with a plasmid encoding a monoFc, DBP Subdomain 3 (sequence as per UniProt P22290 PVDR residues P387-S508), and a C-terminal c-tag. The monoFc was cleaved using TEV protease and sd3 was purified by affinity chromatography (c-tag) and size exclusion chromatography. Plates were washed with washing buffer composed of PBS containing 0.05% TWEEN® 20 (P1379, Sigma-Aldrich) and blocked with 100µL of Starting Block™ T20 (37538, ThermoFisher Scientific). After removing the blocking buffer, standard curve and internal controls were diluted in blocking buffer using a pool of high-titre vaccinee plasma or serum specific to each antigen and isotype or subclass being tested, and 50µL of each dilution was added to the plate in duplicate. Test samples were diluted in blocking buffer to a minimum dilution of 1:50 (or 1:100 for total IgG) and 50µL was added in triplicate. Plates were incubated for 2h at 37°C (or 20°C for total IgG) and washed in washing buffer. An alkaline phosphatase-conjugated secondary antibody was diluted at the manufacturer’s recommended minimum dilution for ELISA in blocking buffer. The antibody used was dependent on the isotype or subclass being assayed and were as follows: IgG-AP (A3187, Thermo Scientific), IgG1 Fc-AP (9054-04, Southern Biotech), IgG3 Hinge-AP (9210-04, Southern Biotech), IgG4 Fc-AP (9200-04, Southern Biotech), IgA-AP (2050-04, Southern Biotech), IgA1-AP (9130-04, Southern Biotech), and IgM-AP (2020-04, Southern Biotech). Then, 50µL of the secondary antibody dilution was added to each well of the plate and incubated for 1h at 37°C (or 20°C for total IgG). Plates were developed using PNPP alkaline phosphatase substrate (N2765, Sigma-Aldrich) for 1-4h at 37°C (or approximately 15 minutes at 20°C for total IgG). Optical density at 405 nm was measured using an ELx808 absorbance reader (BioTek) until the internal control reached an OD405_nm_ of 1. The reciprocal of the internal control dilution giving an OD405_nm_ of 1 was used to assign an AU value of the standard. Gen5 ELISA software v3.04 (BioTek) was used to convert the OD405_nm_ of test samples into AU values by interpolating from the linear range of the standard curve fitted to a four-parameter logistics model. Any samples with an OD405_nm_ below the linear range of the standard curve at the minimum dilution tested were assigned a minimum AU value according to the lower limit of quantification of the assay. For the assessment of IgG2 and IgA2 responses, no anti-DBPRII IgG2 or IgA2 samples were available for standard curve generation. Responses were measured on plates coated with 5µg/mL DBPRII. Four wells were also coated with RH5.1 protein for development control wells. Each sample was tested in duplicate with six negative control serum samples and two development control serum samples on the RH5.1 coated wells from a previous RH5.1/AS01 vaccine trial (4). Secondary antibodies used were IgG2 Fd-AP (9080-04) and IgA2-AP (9140-04). The assay was carried out as above and plates were developed for 2-4h at 37°C.

For the RH5 clinical trial, serum antibody levels to full-length RH5 protein (RH5.1) were assessed by standardised ELISA methodology as previously described (4, 21). In brief, the reciprocal of the test sample dilution giving an optical density of 1.0 at 405nm (OD405_nm_) was used to assign an ELISA unit value of the standard. The standard curve and Gen5 software v3.04 (Agilent) were then used to convert the OD405_nm_ of test samples to arbitrary units (AU). Responses are reported in µg/mL using a conversion factor from AU generated by calibration-free concentration analysis (CFCA), as previously reported (21).

### Bone marrow aspirate processing and ELISPOTs

A single 10mL bone marrow aspirate was collected in EDTA per vaccinee in the RH5 trial. Bone marrow mononuclear cells (BMMNC) were purified from aspirates by density centrifugation on Lymphoprep (1114545, Axis Shield) following passage through a 70µm nylon cell strainer (542070, Greiner Bio-One Ltd). BMMNC were cryopreserved for future use in FCS (S1810, Biosera) with 10% DMSO (D2650, Sigma). Samples were subsequently thawed and enriched for B cells using a Pan B Cell Enrichment Kit (19554, Stemcell) for the detection of RH5-specific plasma cells with an antibody-secreting cell (ASC) ELISPOT. In brief, enriched bone marrow B cells were aliquoted onto MAIP ELISpot Plates (MAIPS4510, Millipore) coated with 5µg/mL RH5 protein, PBS (negative control), or 50µg/mL polyvalent goat anti-human immunoglobulin (positive control; H1700, Caltag). Plates were incubated for 16-18h at 37°C prior to cell removal and incubation with anti-human IgG conjugated to alkaline phosphatase (γ-chain specific; 401442, Calbiochem). Finally, plates were developed with BCIP/NBT (M0711A, Europa Bioproducts) and read on an AID ELISPOT Plate Reader (AID). Note, for consistency, all ASCs are referred to as plasma cells throughout this report.

### *In vivo* growth inhibition

*In vivo* growth inhibition (IVGI) has been reported elsewhere for the DBPRII trial (11). In brief, vaccinees underwent controlled human malaria blood-stage infection 2-4 weeks after their final vaccination and in parallel with unvaccinated infectivity controls. Controlled blood-stage infection was initiated by intravenous injection of blood infected with the PvW1 clone of *P. vivax*. Participant blood parasitemia was measured in real time by qPCR of the 18S ribosomal RNA gene (22). IVGI was calculated for each vaccinee as the percentage reduction in parasite multiplication rate (PMR) relative to the mean PMR of the unvaccinated controls. PMR was modelled for each vaccinee based on log_10_ transformed qPCR data of the *P. vivax* 18S ribosomal RNA gene, using a mean of three replicate qPCR results for each vaccinee per time point (11, 22, 23). Further details of the IVGI/PMR methodology in this trial can be found in the Supplementary Appendix of the primary trial report (11).

### Quantification and statistical analyses

Comparisons were performed between regimens with (two-tailed) Mann-Whitney tests or a Kruskal-Wallis test with Dunn’s correction for multiple comparisons (GraphPad Prism v9). A *p*-value of < 0.05 was considered statistically significant.

Specifics of the CITRUS analyses are outlined in further detail below. For all data, relevant statistical tests and sample sizes are specified in the figure legends.

### Clustering of B cell flow cytometry data with CITRUS

Raw fcs files with file-internal compensation (i.e. acquisition-defined) from the B cell flow cytometry assays with DBPRII or RH5 trial samples were uploaded separately into Cytobank. Live single (B cell-enriched) lymphocytes were gated and then analysed with CITRUS using equal event sampling. All fluorophore channels were used for clustering with the exception of the probes and the viability stain. All groups and time points were run in a single CITRUS analysis per trial. Median expression values for each fluorophore for each CITRUS-defined cluster were exported per sample. Average expression values across all samples for each fluorophore were then calculated per cluster to define FlowJo gating strategies. Clusters with shared gating strategies were combined into new populations (**Table 2**) for re-analysis in FlowJo. Ki67 was included in the CITRUS clustering, but median expression values did not facilitate defining a dichotomous grating strategy and, thus, Ki67 was not utilised in the population definitions. Samples were excluded from analysis if there were <50 cells in the parent population.

## Data availability statement

The original contributions presented in the study are included in the article/**Supplementary Materials**, further inquiries can be directed to the corresponding author/s.

## Ethics statement

The studies involving humans were approved by either the UK National Health Service Research Ethics Services (REC; references 19/SC/0193 and 19/SC/0330) and the UK Medicine and Healthcare products Regulatory Agency (MHRA; reference CTA 21584/0414/001-0001 and CTA 21584/0418/001-0001), or by the Tanzanian Medicines and Medical Devices Authority (reference TMDA0020/CTR/0006/01), the National Institute for Medical Research (references NIMR/HQ/R.8a/Vol.IX/3537 and NIMR/HQ/R.8c/Vol.1/1887), the Ifakara Health Institute Institutional Review Board (reference IHI/IRB/No:49-2020), and the Oxford Tropical Research Ethics Committee (reference 9-20). The studies were conducted in accordance with the local legislation and institutional requirements. The participants provided their written informed consent to participate in this study.

## Author contributions

CN led the study. AO, AM, and SD were chief, principal, and lead investigators in the clinical trials. JB, SS, CM, KK, MH, AL, WK, IM, KM, MB, HD, LK, NE, SR, and CN performed experiments and/or oversaw critical sample processing. JB, SS, NE, and CN analysed and/or reviewed data. VC, PM, and CC contributed to the PvDBPII vaccine. JB and CN wrote the manuscript. All authors contributed to the article and approved the submitted version.
