# Supplementary material for: Analyses of human vaccine-specific circulating and bone marrow-resident B cell populations reveal benefit of delayed vaccine booster dosing with blood-stage malaria antigens
Source: Front Immunol. 2024 Jan 17;14:1193079. doi: 10.3389/fimmu.2023.1193079 (PMC10827869; doi:10.3389/fimmu.2023.1193079)
Supplement: Supplementary file 1 [file DataSheet_1.docx]

## Supplementary Figures


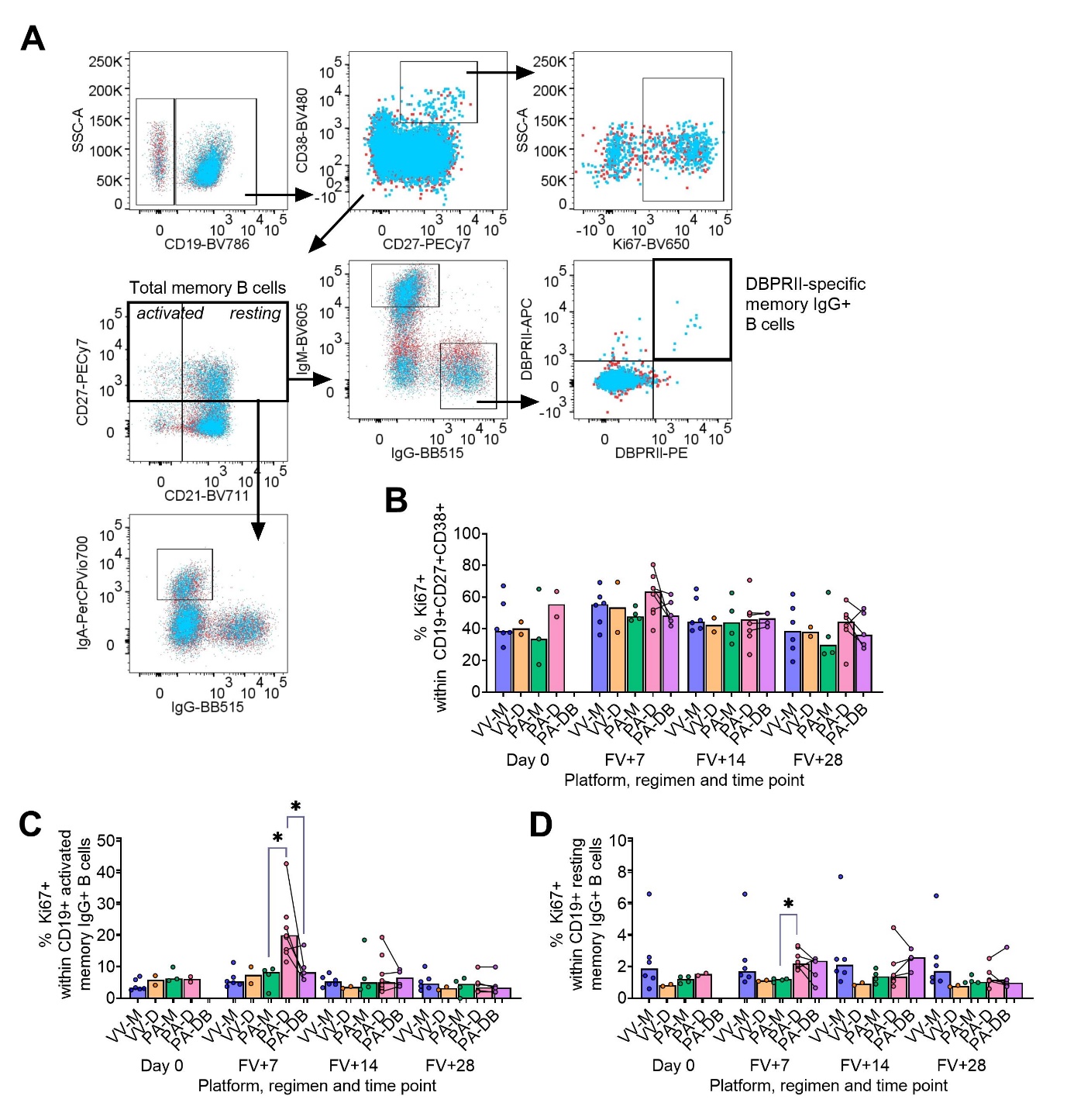


### Supplementary Figure 1. DBPRII B cell gating strategy and expression of proliferation marker Ki67.

PBMC from pre-vaccination (Day 0) and post-final vaccination (FV) time points were analysed for B cell responses by flow cytometry. (**A**) Gating strategy shows identification of CD19+ B cells within live single (B cell-enriched) lymphocytes, and definition of CD38+CD27+ plasma cells within this population. Total memory cells are defined as CD27+ non-plasma cells (following use of a NOT gate to exclude plasma cells; indicated with thick black box); activated and resting memory B cells are more specifically categorised as CD21-CD27+ or CD21+CD27+, respectively. IgG+, IgM+ or IgA+ populations are subsequently gated within total memory cells. Proliferating (Ki67+) or vaccine-specific (those co-staining with DBPRII-PE and DBPRII-APC probes as indicated by thick black box) cells are defined within the plasma cell or isotype-specific memory B cell populations. Example shows Ki67 expression of plasma cells and DBPRII-specific gating on memory IgG+ B cells. An FV+14 sample (blue) is overlaid on a matched Day 0 sample (red) for all plots. Frequencies of Ki67+ cells shown within plasma cells (**B**), activated IgG+ memory B cells (**C**), and resting IgG+ memory B cells (**D**). VV-M = ChAd63-MVA viral vector monthly dosing; VV-D ChAd63-MVA delayed booster dosing; PA-M = protein/adjuvant monthly dosing; PA-D = protein/adjuvant delayed booster dosing; PA-DB = protein/adjuvant delayed booster dosing with extra booster. Post-vaccination comparisons were performed between protein/adjuvant dosing regimens by Kruskal Wallis test with Dunn’s correction for multiple comparisons. Sample sizes for all assays were based on sample availability; each circle represents a single sample. VV-M/VV-D/PA-M/PA-D/PA-DB: Day 0 = 6/2/3-4/2/na, FV+7 = 6/2/4/8/5, FV+14 = 6/2/4/8/4, FV+28 = 6/2/4/6/5. PA-D vaccinees returning in the PA-DB group are connected by lines. Bars represent medians. * *p* < 0.05.


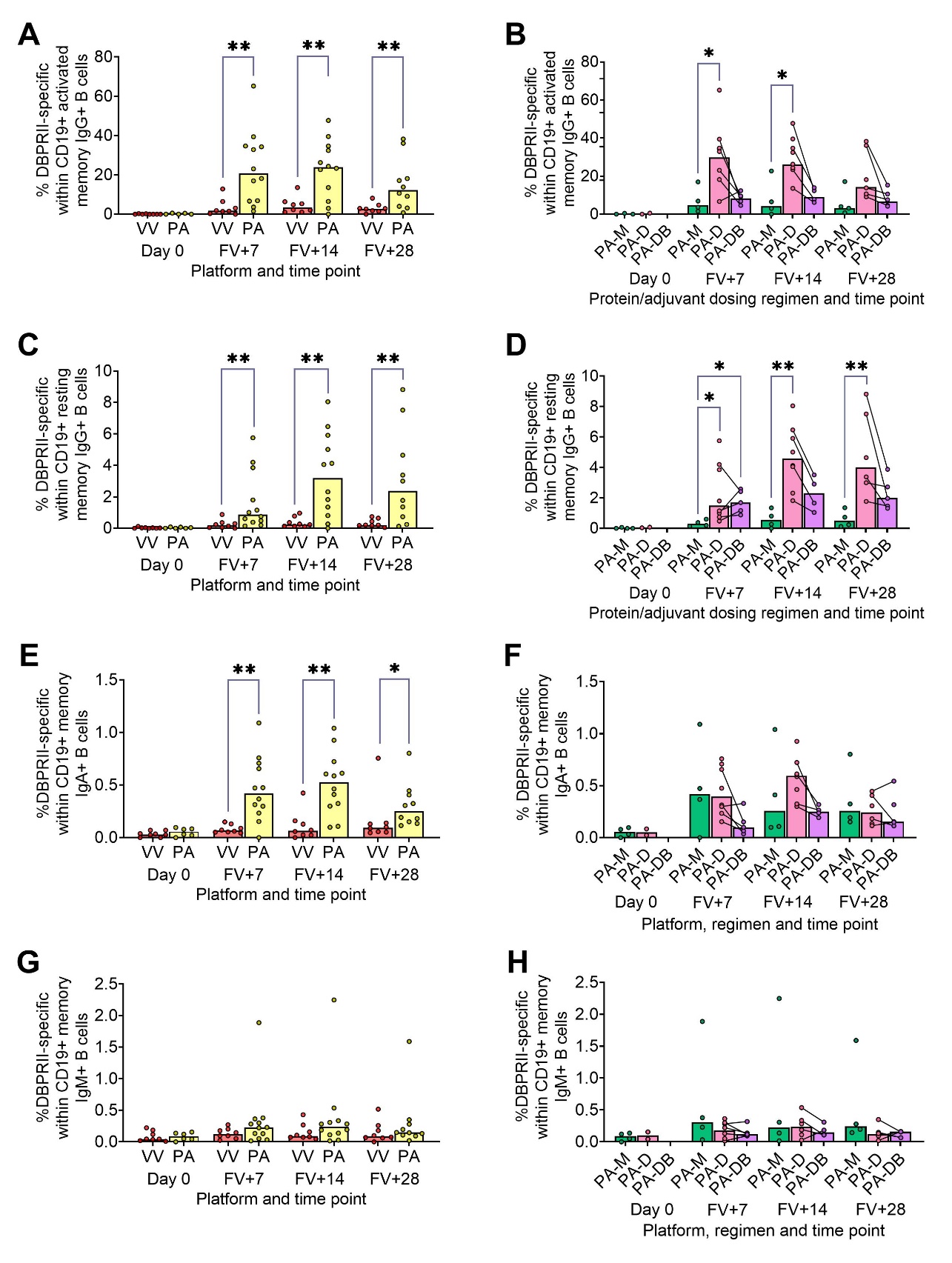


### Supplementary Figure 2. Extended DBPRII-specific memory B cell responses.

PBMC from pre-vaccination (Day 0) and post-final vaccination (FV) time points were analysed for B cell responses by flow cytometry; gating strategies are as described in Methods and **Supplementary Figure 1**. Frequencies of DBPRII-specific B cells – identified by probe staining – were compared within activated memory IgG+ B cells (**A-B**), resting memory IgG+ B cells (**C-D**), total memory IgA+ B cells (**E-F**), and total memory IgM+ B cells (**G-H**) between vaccine platforms (**A, C, E, G**) or protein/adjuvant dosing regimens (**B, D, F, H**). VV = ChAd63-MVA viral vectors [monthly and delayed dosing]; PA = PvDBPII protein/adjuvant [PA-M and PA-D]; PA-M = PvDBPII protein/adjuvant monthly dosing; PA-D = PvDBPII protein/adjuvant delayed booster dosing; PA-DB = PvDBPII protein/adjuvant delayed booster dosing with extra booster. Post-vaccination comparisons were performed between DBPRII platforms (**A, C, E, G**) with Mann-Whitney U tests, or between PvDBPII protein/adjuvant dosing regimens by Kruskal Wallis test with Dunn’s correction for multiple comparisons (**B, D, F, H**). Sample sizes for all assays were based on sample availability; each circle represents a single sample. (**A, C, E, G**) VV/PA: Day 0 = 8/5-6, FV+7 = 8/12, FV+14 = 8/12, FV+28 = 8/10. (**B, D, F, H**) PA-M/PA-D/PA-DB: Day 0 = 3-4/2/na, FV+7 = 4/8/5, FV+14 = 4/8/4, FV+28 = 4/6/5. PA-D vaccinees returning in the PA-DB group are connected by lines. Bars represent medians. * *p* < 0.05, ** *p* < 0.01.


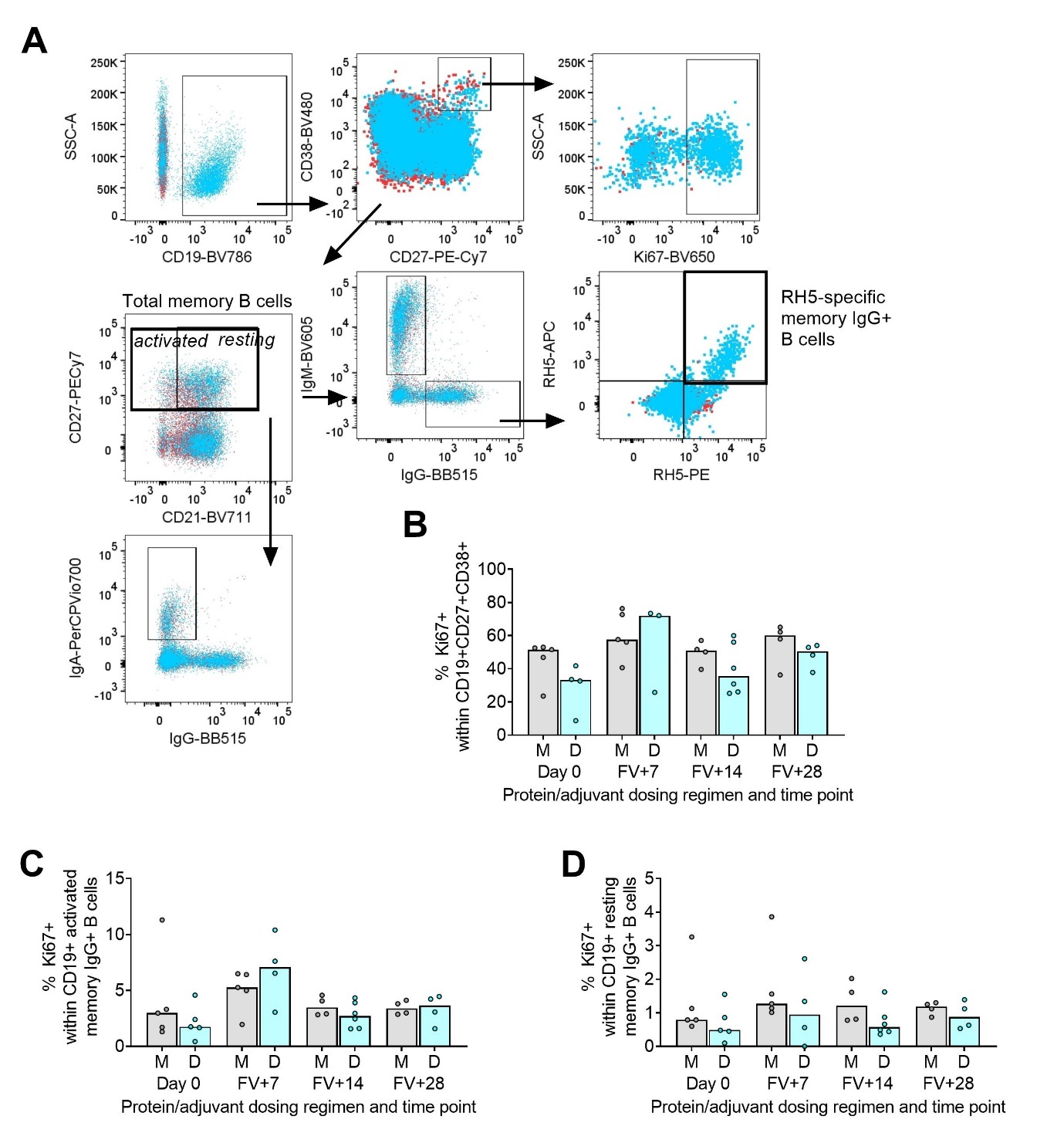


### Supplementary Figure 3. RH5 B cell gating strategy and expression of proliferation marker Ki67.

PBMC from pre-vaccination (Day 0) and post-final vaccination (FV) time points were analysed for B cell responses by flow cytometry. (**A**) Gating strategy shows identification of CD19+ B cells within live single (B cell-enriched) lymphocytes, and definition of CD38+CD27+ plasma cells within this population. Total memory cells are defined as CD27+ non-plasma cells (using a NOT gate to exclude plasma cells; indicated with thick black box); activated and resting memory B cells are more specifically categorised as CD21-CD27+ or CD21+CD27+, respectively. IgG+, IgM+ or IgA+ populations are subsequently gated within total memory cells. Proliferating (Ki67+) or vaccine-specific (those co-staining with RH5-PE and RH5-APC probes as indicated by thick black box) cells are defined within the plasma cell or isotype-specific memory B cell populations. Example shows Ki67 expression of plasma cells and RH5-specific gating on memory IgG+ B cells. An FV+14 sample (blue) is overlaid on a matched Day 0 sample (red) for all plots. Frequencies of Ki67+ cells shown within plasma cells (**B**), activated IgG+ memory B cells (**C**), and resting IgG+ memory B cells (**D**). M = RH5.1/adjuvant monthly dosing; D = RH5.1/adjuvant delayed booster dosing. Post-vaccination comparisons were performed between dosing regimens with Mann-Whitney U tests. Sample sizes for all assays were based on sample availability; each circle represents a single sample. (**B-D**) M/D: Day 0 = 5/4-5, FV+7 = 5/3-4, FV+14 = 4/6, FV+28 = 4/4. Bars represent medians.

###
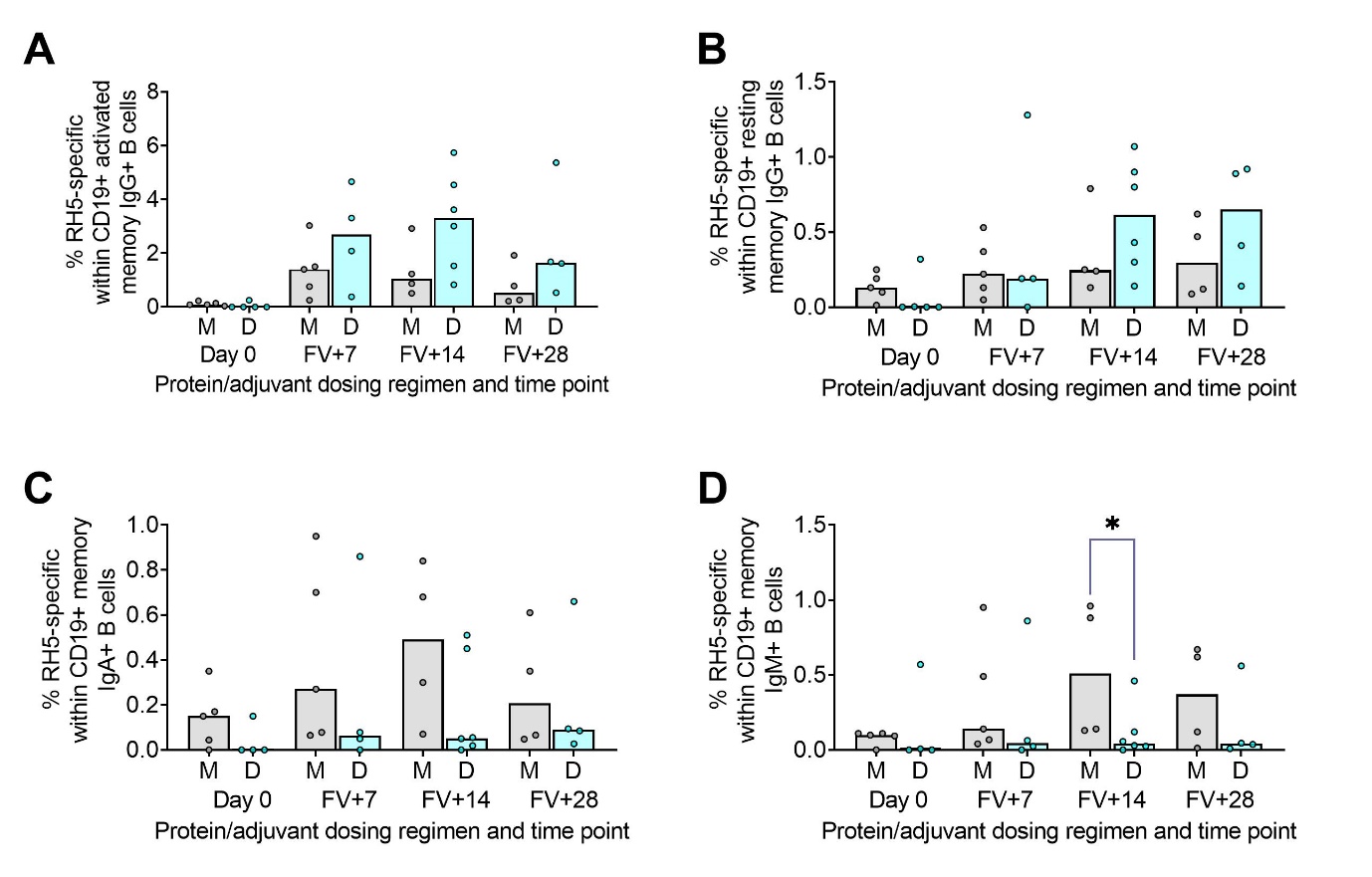


### Supplementary Figure 4. Extended RH5-specific memory B cell responses.

PBMC from pre-vaccination (Day 0) and post-final vaccination (FV) time points were analysed for B cell responses by flow cytometry; gating strategies are as described in Methods and **Supplementary Figure 3**. Frequencies of RH5-specific B cells – identified by probe staining – were compared within activated memory IgG+ B cells (**A**), resting memory IgG+ B cells (**B**), total memory IgA+ B cells (**C**), and total memory IgM+ B cells (**D**) between dosing regimens. M = RH5.1/adjuvant monthly dosing; D = RH5.1/adjuvant delayed booster dosing. Post-vaccination comparisons were performed between dosing regimens with Mann-Whitney U tests. Sample sizes for all assays were based on sample availability; each circle represents a single sample. (**B-D**) M/D: Day 0 = 5/4-5, FV+7 = 5/4 FV+14 = 4/6, FV+28 = 4/4. Bars represent medians. *** *p* < 0.05.

###
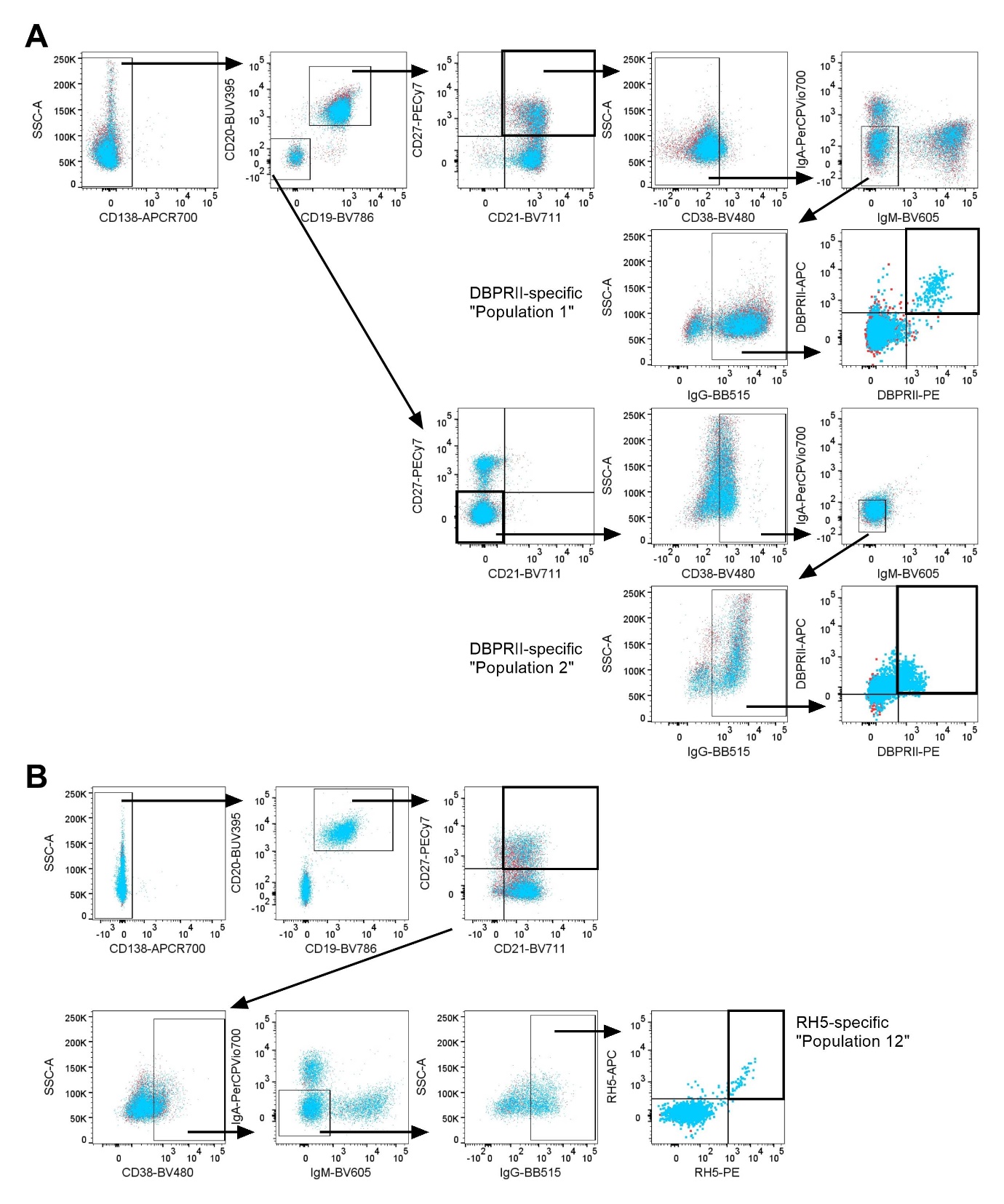
Supplementary Figure 5. Gating strategies for key agnostically defined B cell populations via CITRUS.

CITRUS was run on single live (B cell-enriched) lymphocyte flow cytometry fcs files to agnostically define the main B cell populations within either DBPRII or RH5 trial samples. Median marker expression within each cluster was used to define gating strategies for B cell populations in FlowJo, which were re-analysed for DBPRII- or RH5-specific responses through probe staining. (**A**) Gating strategy shows identification of “Population 1” (CD19+CD20+CD21+CD27+CD138-CD38-IgM-IgA-IgG+) and “Population 2” (CD19-CD20-CD21-CD27-CD138-CD38+IgM-IgA-IgG+;) within the DBPRII trial and DBPRII-specific cells within these populations. (**B**) Gating strategy shows identification of “Population 12” (CD19+CD20+CD21+CD27+ CD138-CD38+IgA-IgM-IgG+) within the RH5 trial. An FV+14 sample (blue) is overlaid on a matched Day 0 sample (red) for all plots. See **Table 2** for a full list of populations defined via CITRUS.


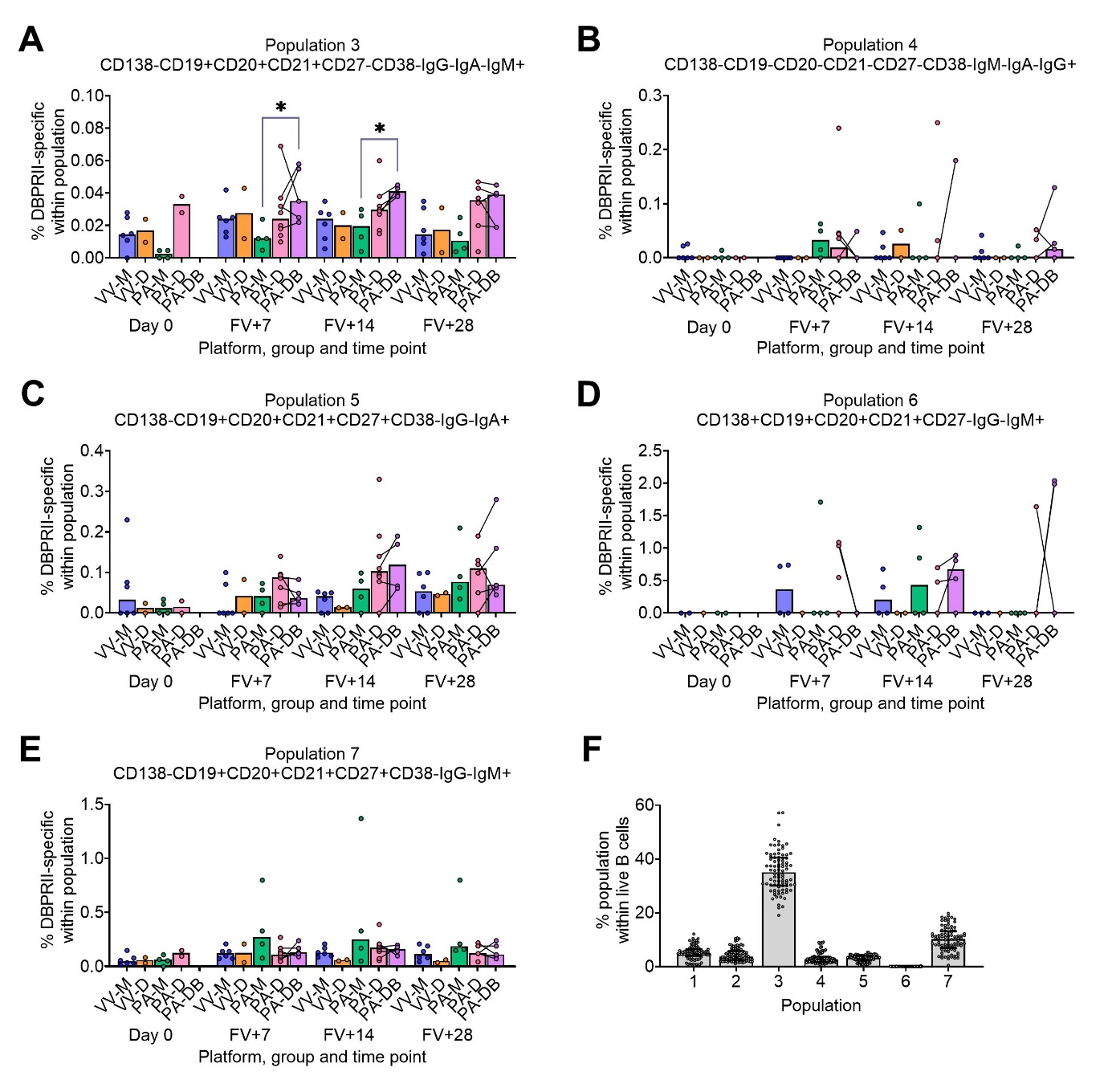


### Supplementary Figure 6. DBPRII-specific responses within agnostically defined B cell populations.

CITRUS was run on single live (B cell-enriched) lymphocyte flow cytometry fcs files to agnostically define the main B cell populations within DBPRII trial samples. Median marker expression within each cluster was used to define gating strategies for B cell populations in FlowJo, which were re-analysed for DBPRII-specific responses through probe staining (**A-E**). Population definitions are annotated on individual figures. (**F**) Frequencies of each population within single live B cells (enriched from lymphocytes; see also **Table 2**) of all samples. VV-M = ChAd63-MVA viral vector monthly dosing; VV-D ChAd63-MVA delayed booster dosing; PA-M = PvDBPII protein/adjuvant monthly dosing; PA-D = PvDBPII protein/adjuvant delayed booster dosing; PA-DB = PvDBPII protein/adjuvant delayed booster dosing with extra booster. FV = final vaccination. Post-vaccination comparisons were performed between PvDBPII protein/adjuvant dosing regimens by Kruskal Wallis test with Dunn’s correction for multiple comparisons (**A-E**). Sample sizes for all assays were based on sample availability; each circle represents a single sample. (**A-C, E**) VV-M/VV-D/PA-M/PA-D/PA-DB: Day 0 = 6/2/4/2/na, FV+7 = 6/2/4/8/5, FV+14 = 6/2/4/8/4, FV+28 = 6/2/4/6/5. PA-D vaccinees returning in the PA-DB group are connected by lines. (**D**) VV-M/VV-D/PA-M/PA-D/PA-DB: Day 0 = 2/1/2/0/na, FV+7 = 4/1/4/8/4, FV+14 = 4/2/4/8/4, FV+28 = 3/1/4/5/5. (**F**) *n* = 86 for all populations. Bars represent medians. * *p* < 0.05.

*
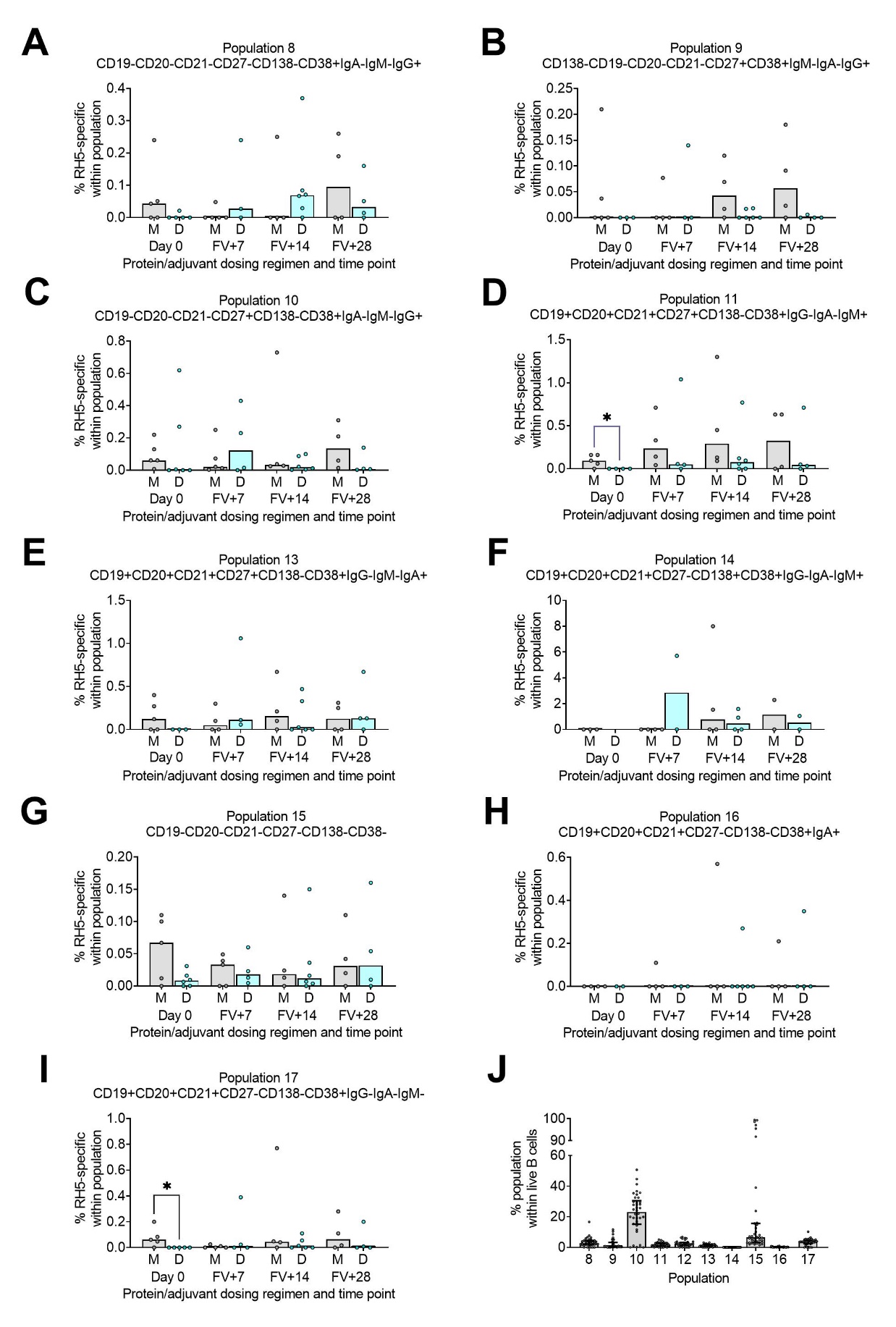
*

### Supplementary Figure 7. RH5-specific responses within agnostically defined B cell populations.

CITRUS was run on single live (B cell-enriched) lymphocyte flow cytometry fcs files to agnostically define the main B cell populations within RH5 trial samples. Median marker expression within each cluster was used to define gating strategies for B cell populations in FlowJo, which were re-analysed for RH5-specific responses through probe staining (**A-I**). Population definitions are annotated on individual figures. (**J**) Frequencies of each population within single live B cells (enriched from lymphocytes; see also **Table 2**) of all samples. M = RH5.1/adjuvant monthly dosing; D = RH5.1/adjuvant delayed booster dosing. FV = final vaccination. Post-vaccination comparisons were performed between dosing regimens with Mann-Whitney U tests (**A-I**). Sample sizes for all assays were based on sample availability; each circle represents a single sample. (**A-G, I**) M/D Day 0 = 4-5/2-6, FV+7 = 4-5/3-4, FV+14 = 4/6, FV+28 = 4/4. (**H**) M/D Day 0 = 3/0, FV+7 = 4/2, FV+14 = 4/4, FV+28 = 2/2. (**J**) *n* = 38 for all populations. Bars represent medians. * *p* < 0.05.


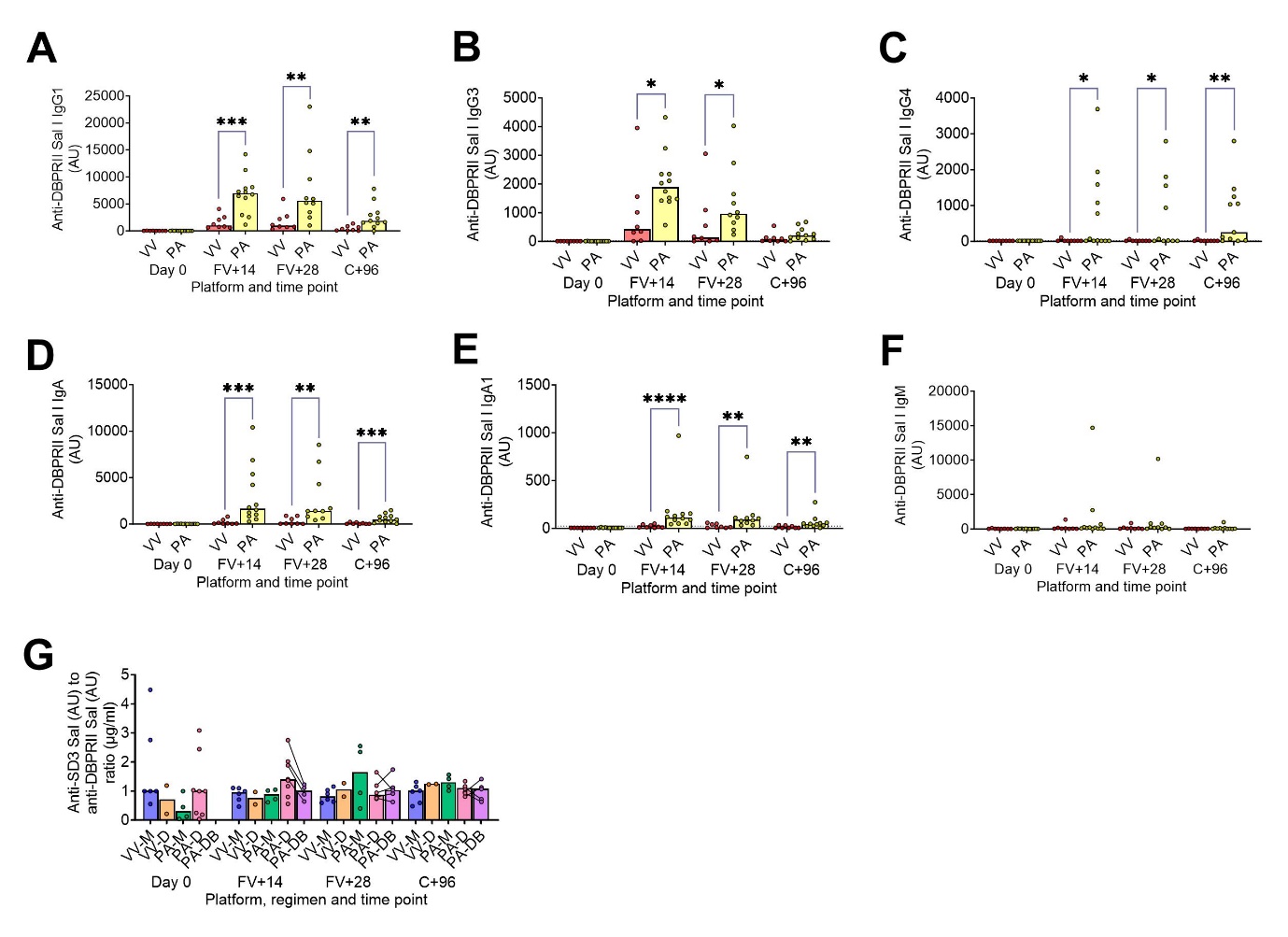


Supplementary Figure 8. DBPRII-specific peak antibody responses and serum maintenance.

Standardised ELISAs were developed to report anti-DBPRII specific or anti-subdomain 3 (sd3) antibody responses in pre-vaccination (Day 0) and post-final vaccination (FV) serum samples. Responses were compared between vaccine platforms for IgG1 (**A**), IgG3 (**B**), IgG4 (**C**), IgA (**D**), IgA1 (**E**), and IgM (**F**). IgG2 and IgA2 responses were below the limit of detection (not shown). The ratio of anti-sd3 to anti-DBPRII was also calculated for total IgG (**G**). VV = ChAd63-MVA viral vectors; PA = PvDBPII protein/adjuvant [PA-M and PA-D]; VV-M = ChAd63-MVA viral vector monthly dosing [monthly and delayed dosing]; VV-D ChAd63-MVA delayed booster dosing; PA-M = protein/adjuvant monthly dosing; PA-D = protein/adjuvant delayed booster dosing; PA-DB = protein/adjuvant delayed booster dosing with extra booster. C+96 = 96 days after controlled human malaria infection (approximately 16 weeks after FV). Post-vaccination comparisons were performed between platforms (**A-F**) with Mann-Whitney U tests, or between protein/adjuvant dosing regimens by Kruskal Wallis test with Dunn’s correction for multiple comparisons (**G**). Sample sizes for all assays were based on sample availability; each circle represents a single sample. (**A-F**) VV/PA: Day 0 = 8/12, FV+14 = 8/12, FV+28 = 8/10. (**G**) VV-M/VV-D/PA-M/PA-D/PA-DB: Day 0 =6/2/4/8/na, FV+14 = 6/2/4/8/4, FV+28 = 6/2/4/6/5, C+96 = 6/2/4/7/5. PA-D vaccinees returning in the PA-DB group are connected by lines. Bars represent medians. * *p* < 0.05, ** *p* < 0.01, *** *p* < 0.001, **** *p* < 0.0001.

###
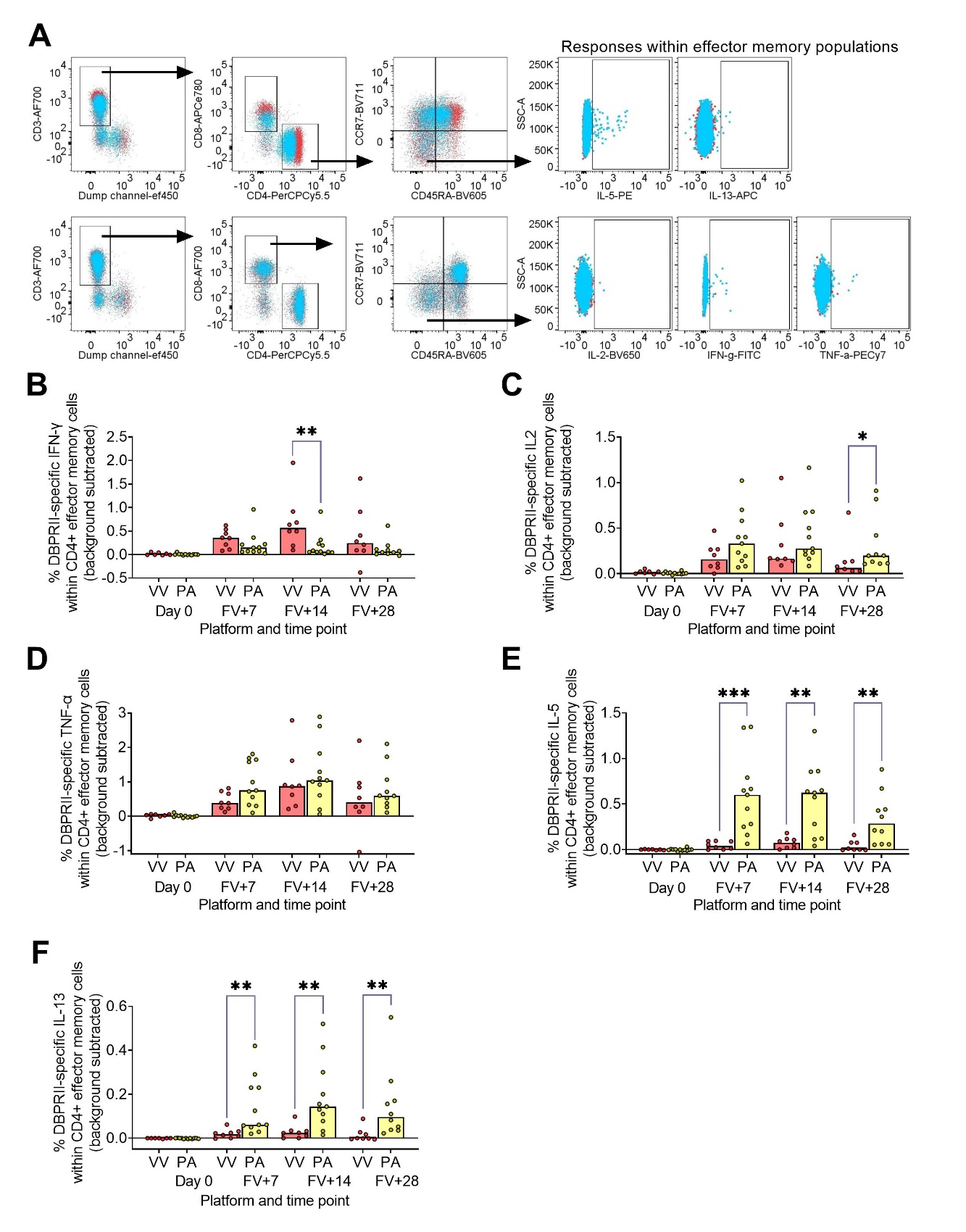
 Supplementary Figure 9. DBPRII-specific T cell gating strategy and CD4+ effector memory individual cytokine responses. PBMC from pre-vaccination (Day 0) and post-final vaccination (FV) time points were analysed for T cell responses by intracellular cytokine staining. (**A**) Gating strategy shows identification of live T cells within single lymphocytes [dump channel includes viability stain, anti-CD14, and anti-CD19; see Methods), and definition of CD4+ and CD8+ T cells within this population. Effector memory CD4+ or CD8+ T cells are identified as CD45RA-CCR7-. Finally, DBPRII-specific T cell responses are defined through the detection of intracellular Th2 cytokines (top row; IL-5, IL-13;) or Th1 cytokines (bottom row; IL-2, IFN-γ, TNF-α) following DBPRII peptide pool stimulation. An FV+7 sample (blue) is overlaid on a matched Day 0 sample (red) for all plots (CD4+ effector memory from protein/adjuvant vaccinee used as top row Th2 example; CD8+ effector memory from viral vector vaccinee used as bottom row Th1 example). DBPRII-specific effector memory CD4+ or CD8+ T cells are reported as frequencies producing cytokines in response to peptide stimulation after background subtraction of cytokine-positive cells in matched samples cultured with media alone. Using an ‘OR’ gate, responses were reported for all cytokines (cells producing any of IL-5, IL-13, IL-2, IFN-γ, or TNF-α), Th1 cytokines (IL-2, IFN-γ or TNF-α only), or Th2 cytokine (IL-5 or IL-13 only; see **Figure 4**). CD4+ effector memory T cells producing IFN-γ (**B**), IL-2 (**C**), TNF-α (**D**), IL-5 (**E**) or IL-13 (**F**) were compared between vaccine platforms. VV = ChAd63-MVA viral vectors [monthly and delayed dosing]; PA = PvDBPII protein/adjuvant [PvDBPII protein/adjuvant monthly dosing and PvDBPII protein/adjuvant delayed booster dosing]. Post-vaccination comparisons were performed between DBPRII platforms by Mann Whitney U test. Sample sizes for all assays were based on sample availability; each circle represents a single sample. VV/PA: Day 0 = 7/12, FV+7 = 8/11, FV+14 = 8/11, FV+28 = 8/10. Bars represent medians. * p < 0.05, ** p < 0.01, *** p < 0.001.


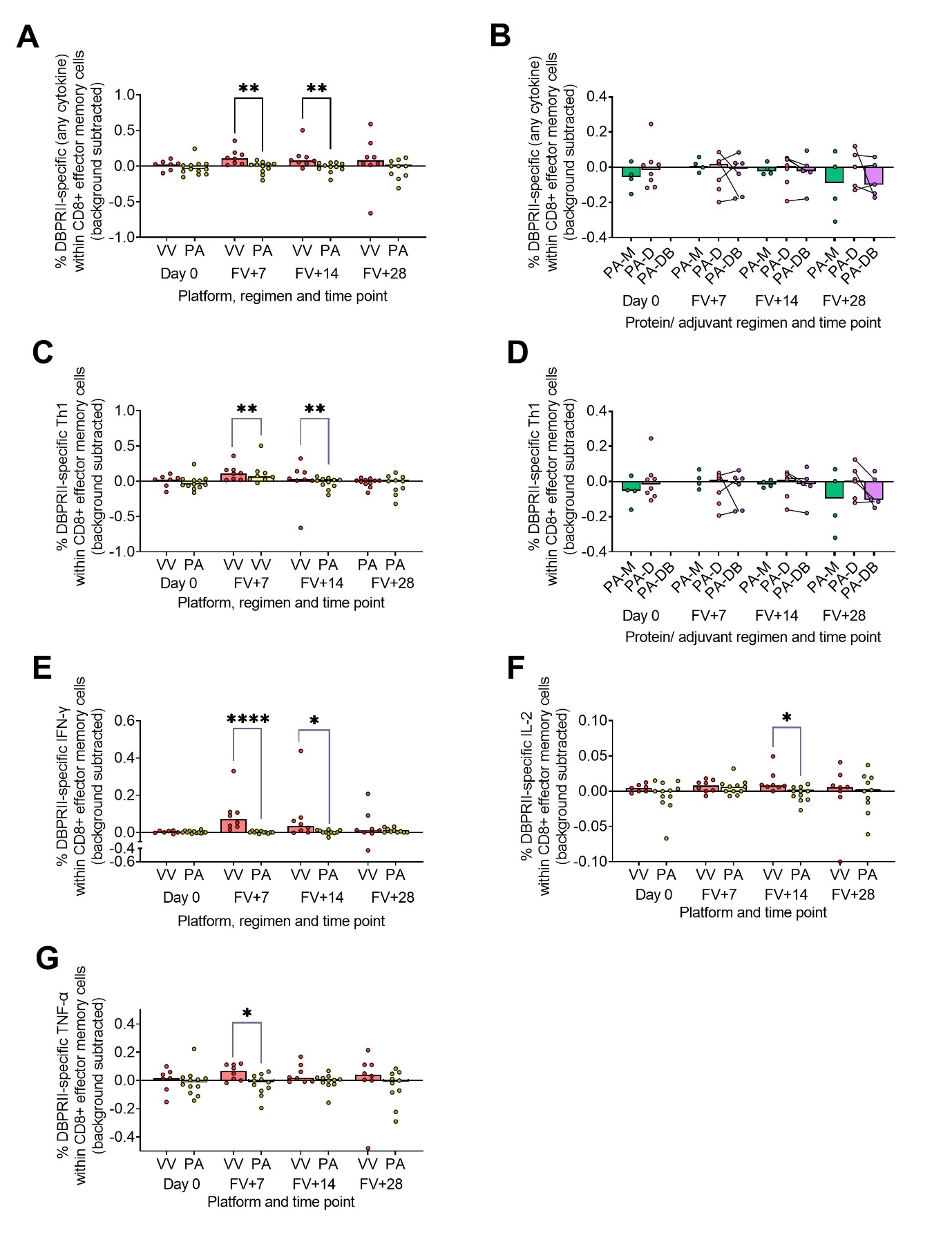


### Supplementary Figure 10. DBPRII-specific CD8+ T cell effector memory responses.

PBMC from pre-vaccination (Day 0) and post-final vaccination (FV) time points were analysed for T cell responses by intracellular cytokine staining; gating strategies are as described in Methods and **Supplementary Figure 9**. In brief, DBPRII-specific effector memory CD8+ T cells are reported as frequencies producing cytokines in response to peptide stimulation after background subtraction of cytokine-positive cells in matched samples cultured with media alone. DBPRII-specific responses were defined by production of any cytokine [IL-2, IL-5, IL-13, IFN-γ, TNF-α] (**A-B**), any Th1 cytokine [IL-2, IFN-γ, TNF-α] (**C-D**), or individual Th1 cytokines: IFN- γ (**E),** IL-2 **(F)** and TNF-α **(G)**. These CD8+ effector memory T cells were compared between vaccine platforms (**A, C, E-G**) or protein/adjuvant dosing regimens (**B, D**). VV = ChAd63-MVA viral vectors; PA = PvDBPII protein/adjuvant [PA-M and PA-D]; PA-M = PvDBPII protein/adjuvant monthly dosing; PA-D = PvDBPII protein/adjuvant delayed booster dosing; PA-DB = PvDBPII protein/adjuvant delayed booster dosing with extra booster. Post-vaccination comparisons were performed between DBPRII platforms by Mann Whitney U test (**A, C, E-G**), or protein/adjuvant dosing regimens by Kruskal Wallis test with Dunn’s correction for multiple comparisons (**B, D**). Sample sizes for all assays were based on sample availability; each circle represents a single sample. (**A, C, E-G**) VV/PA: Day 0 = 7/12, FV+7 = 8/11, FV+14 = 8/11, FV+28 = 8/10. (**B, D**) PA-M/PA-D/PA-DB: Day 0 = 4/8/na, FV+7 = 4/7/6, FV+14 = 4/7/5, FV+28 = 4/6/5. PA-D vaccinees returning in the PA-DB group are connected by lines. Bars represent medians. * *p* < 0.05, ** *p* < 0.01, **** *p* < 0.0001.
